# Corticotropin-releasing factor neurons in the bed nucleus of the stria terminalis exhibit sex-specific pain encoding in mice

**DOI:** 10.1038/s41598-021-91672-8

**Published:** 2021-06-14

**Authors:** Waylin Yu, Christina M. Caira, Natalia del R. Rivera Sanchez, Garrett A. Moseley, Thomas L. Kash

**Affiliations:** 1 Department of Pharmacology, School of Medicine, University of North Carolina At Chapel Hill, CB 7178 Thurston Bowles Building, 104 Manning Drive, Chapel Hill, NC 27599 USA; 2 Bowles Center for Alcohol Studies, University of North Carolina At Chapel Hill, Chapel Hill, NC 27599 USA; 3 Curriculum in Pharmacology, School of Medicine, University of North Carolina At Chapel Hill School of Medicine, Chapel Hill, NC 27599 USA

**Keywords:** Neuroscience, Neuronal physiology, Sensory processing, Stress and resilience

## Abstract

The bed nucleus of the stria terminalis (BNST) plays an emerging role in pain regulation. Pharmacological studies have found that inhibiting corticotropin-releasing factor (CRF) signaling in the BNST can selectively mitigate the sensory and affective-motivational components of pain. However, mechanistic insight on the source of CRF that drives BNST responses to these harmful experiences remains unknown. In the present study, we used a series of genetic approaches to show that CRF in the BNST is engaged in the processing and modulation of pain. We conducted cell-type specific in vivo calcium imaging in CRF-Cre mice and found robust and synchronized recruitment of BNST^CRF^ neurons during acute exposures to noxious heat. Distinct patterns of recruitment were observed by sex, as the magnitude and timing of heat responsive activity in BNST^CRF^ neurons differed for male and female mice. We then used a viral approach in Floxed-CRF mice to selectively reduce CRF expression in the BNST and found it decreased nociceptive sensitivity for both sexes and increased paw attending for females. Together, these findings reveal that CRF in the BNST influences multiple facets of the pain experience to impact the sex-specific expression of pain-related behaviors.

## Introduction

Pain is a pervasive source of stress that disrupts homeostasis by driving sensory experiences associated with negative emotional states^1^. Corticotropin-releasing factor (CRF), a peptide known for regulating stress-related behaviors, has been hypothesized to play an impactful, albeit understudied, role in pain modulation^15,32,42,45,55,56,68,71,74,79,81,83^. Preclinical studies have highlighted the bed nucleus of the stria terminalis (BNST) as a CRF-enriched structure that significantly influences the emotional aspects of pain^49,50,62^. Bilateral lesions of the BNST suppress pain-related aversion in rodents^14,21–23^, while intraplanar treatment with formalin, a tonic-acting inflammatory agent that exacerbates sensitivity to noxious stimuli, increases CRF release in the BNST and generates conditioned place aversion in a CRF_1_ and CRF_2_ receptor-dependent manner^35^. Notably, no effect of CRF on paw attending behaviors was reported in these studies^35,40^, suggesting that CRF signaling in the BNST contributes to the emotional—but not sensory—components of pain. By contrast, intra-BNST antagonism of CRF_2R_ reduces mechanical and colonic nociception, and CRF_1R_ antagonism attenuates stress-induced hyperalgesia^76,77^, revealing that similar manipulations of CRF signaling can impact sensation when different sets of stimuli are used to drive the pain experience. The discrepancies in CRF contributions to pain sensitivity reported in these studies indicate the need for additional evaluations on the conditions by which CRF signaling in the BNST can alter the sensory and affective-motivational components of pain.

To gain a better understanding of CRF signaling in the BNST and its contributions to pain, two key functional components require clarification. (1) To date, the majority of studies focusing on the BNST and pain have been limited to pharmacological examinations of local CRF receptors, notably lacking insight on the sources of CRF that influence the conditional effects of CRF receptor antagonism on pain^35,57,76,77^. (2) Previous studies were performed exclusively in male subjects, despite evidence that CRF signaling in the BNST drives discrete stress responses in male and female rodents^36,43,72^. Sex differences in pain expression and treatment are well-established in humans^6,58^, but preclinical pain studies have historically excluded female subjects^52^, thus increasing the potential for neglected mechanistic insight. These uncharacterized features of CRF signaling reveal significant gaps in our understanding of how the BNST regulates pain.

In the present study, we sought to determine if the BNST is a source of CRF that influences pain-related behaviors in male and female mice. Given the density of CRF signaling in the BNST, we posit that local production of CRF would be necessary for pain regulation, similar to the function of local CRF receptors^16,18,19,39,51,54,66^. CRF expression in the BNST may additionally contribute to differential expressions of pain in male and female subjects, since sexual dimorphism is a notable feature of the BNST^20,30^ and CRF signaling^4^. To test these hypotheses, we characterized BNST^CRF^ contributions to pain processing and modulation in both sexes by implementing genetically targeted manipulations of the local CRF population. We first used in vivo calcium imaging to examine how BNST^CRF^ neurons encode pain, specifically tracking the endogenous recruitment of cell activity relative to acute noxious heat exposure. We then used a viral approach in Floxed-CRF mice to knock down CRF expression in the BNST and assess pain-related behaviors, allowing us to discern peptide-specific contributions to acute and sustained forms of pain. Our findings demonstrate that CRF neurons in the BNST are activated by the detection of pain and locally produced CRF is necessary for intact behavioral responses to pain. The extent of these effects varies by sex, suggesting that local production of CRF in the BNST makes distinct contributions to pain in male and female mice. By monitoring endogenous BNST^CRF^ neuronal activity and manipulating peptide expression in both sexes, our study highlights the BNST as an important region for sex-dependent pain regulation via local CRF expression.

## Materials and methods

### Animals

Male and female CRF-Cre (*Crh*^IRES-Cre^ from Lowell lab^44^) and Floxed-CRF (*Crh*^lox-lox^ from Zweifel lab^67^) mice (N = 103 total, ≥ 6 weeks of age, age-matched for each experiment, C57BL/6 J background) were bred in-house, group-housed with same-sex littermates, and maintained on a 12-h light/dark cycle (light on at 7:00, light off at 19:00) with rodent chow and water available ad libitum. Subjects were singly housed for imaging experiments to ensure recovery and prevent post-surgical complications. All procedures were approved by the Institutional Animal Care and Use Committee at UNC Chapel Hill and performed in accordance with the NIH Guide for the Care and Use of Laboratory Animals and ARRIVE guidelines^41^.

### Surgeries

Subjects were anesthetized with isoflurane (1–3%) in oxygen (1–2 l/min) and aligned on a stereotaxic frame (Kopf Instruments, Tujunga, CA). All surgeries were conducted using aseptic techniques in a sterile environment. Microinjections were performed with a 1 µl Neuros Hamilton syringe (Hamilton, Reno, NV) and a micro-infusion pump (KD Scientific, Holliston, MA) that infused virus at 100 nl/min. Viruses were administered bilaterally in the dorsal region of BNST (250 nl of AAVDJ-EF1a-DIO-GCaMP6s [UNC Vector Core, Chapel Hill, NC; Lot # AV7438; Titer = 3.1 × 10^12^ infectious units/mL], AAV5-CaMKIIa-Cre-GFP [UNC Vector Core, Chapel Hill, NC; Lot # AV6450; Titer = 5 × 10^12^ infectious units/mL], or AAV5-CaMKIIa-GFP [UNC Vector Core, Chapel Hill, NC; Lot # AV4621; Titer = 4 × 10^12^ infectious units/mL] per injection; relative to bregma: ML ± 0.90 mm, AP + 0.23 mm, DV − 4.35 mm). For experiments that required in vivo calcium imaging, Gradient-index (GRIN) lenses were implanted unilaterally in the right hemisphere approximately 200 µm over the dorsal BNST (relative to bregma: ML ± 0.90 mm, AP 0.23 mm, DV − 4.15 mm) and secured with a dental cement headcap. Baseplates were later added to stabilize the attachment of the miniature microscope (see “*Ca*^*2*+^ *Imaging with Miniature Microscope*” section for details). After surgery, mice were given ad libitum Tylenol water or daily injections of meloxicam (5 mg/kg, subcutaneous [s.c.]) for four consecutive days, then allowed to recover for three weeks or longer before starting experiments.

### Behavioral assays

#### Preliminary habituation

Prior to behavioral testing, subjects experienced 3 days of handling for 5 min each day. Subjects contributing to imaging experiments were given 2 additional days of miniature microscope tethering in their home cage for 15 min. Subjects were then exposed to a battery of behavioral tests in the following order: (i) Open Field, (ii) Elevated Plus Maze, (iii) Hargreaves, (iv) Von Frey, (v) Tail Immersion, (vi) Hot Plate. Each test was separated by at least 24 h to prevent the possibility of impaired test performance.

#### Hargreaves

**Figure 1:**
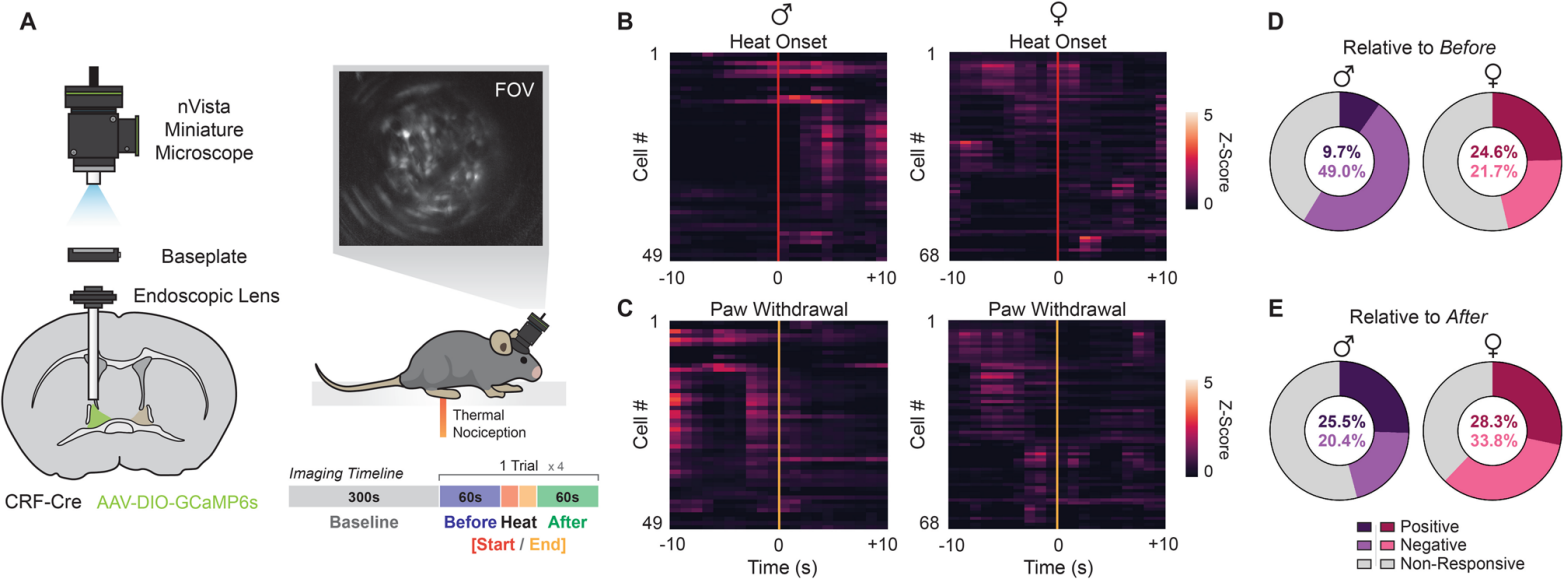
Measurement of Single-Cell Ca^2+^ Activity in BNST^CRF^ Neurons During Pain (**A**) Miniature microscope imaging in BNST^CRF^ neurons. (Left) Diagram of GCaMP6s infusion and endoscopic lens / baseplate implantation in the BNST of CRF-Cre mice. (Upper-Right) Imaging field-of-view (FOV) depicting GCaMP6s expression in BNST^CRF^ neurons. (Lower-Right) Experimental timeline with schematic of BNST^CRF^ imaging during behavioral assessment of thermal nociceptive sensitivity. Epochs relative to noxious heat exposure are color-coded: *Before* (blue), *Heat Start* (red), *Heat End* (orange), and *After* (green). To ensure that epochs represent equal amounts of time on a trial-by-trial basis, epoch lengths were quantitatively defined as half the duration of heat exposure for each specific subject and trial. (**B–C**) Peri-event heatmap of average BNST^CRF^ activity surrounding (**B**) heat onset (red line) and (**C**) paw withdrawal (orange line) for male (left column; n = 49 cells / 7–30 cells per mouse) and female (right column; n = 68 cells / 8–19 cells per mouse) subjects. Lighter shades of color correspond to higher z-scores, a metric indicative of greater changes in Ca^2+^ activity relative to baseline. (**D–E**) Total percentage of pain responsive BNST^CRF^ neurons for male (purple) and female (magenta) subjects across trials. Cells that respond to heat exposure with positive (darker), negative (lighter), and no (grey) changes in z-score were determined relative to the *Before* and *After* epochs, as demarcated by the onset of (**D**) heat exposure [“Relative to *Before*”] and (**E**) paw withdrawal [“Relative to *After*”] respectively (Wilcoxon rank-sum, *P* < 0.05). Data are shown as mean ± SEM. *P < 0.05; **P < 0.01; ***P < 0.001, ****P < 0.0001.

**Figure 2:**
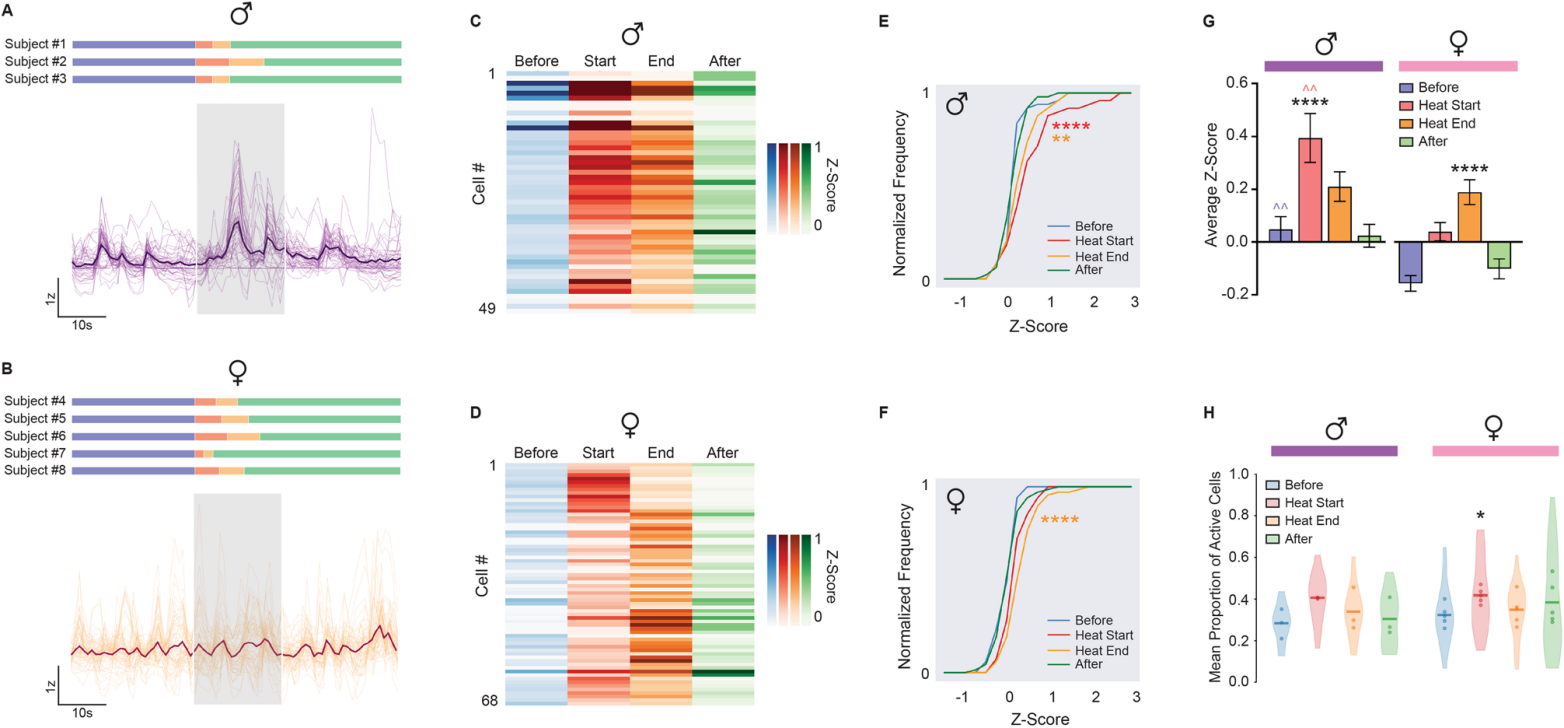
Sex-Dependent BNST^CRF^ Responses to Noxious Heat Exposure by Epoch. (**A–B**) Relative fluorescence change (ΔF/F) of BNST^CRF^ activity during noxious heat exposure. Z-scores of (**A**) male (n = 49 cells / 7–30 cells per mouse) and (**B**) female (n = 68 cells / 8–19 cells per mouse) subjects are represented over time by aligning traces that were averaged across trials to the onset of the heat stimulus. Individual cell activity is indicated in lighter colors, with total average cell activity in darker colors, and maximum heat duration (20 s) in the transparent blue block. Scale bars, x = time (10 s), y = z-score based on relative ΔF/F (1 z-score). Imaging timelines for individual subjects are located above to represent epochs via the color-coding system described in Fig. 1A: *Before* (blue), *Heat Start* (red), *Heat End* (orange), and *After* (green). Similar color designations apply for all panels of Fig. 2. (**C–D**) Heatmap exhibiting the average z-score of individual BNST^CRF^ neurons by epoch for (**C**) male and (**D**) female subjects. Darker shades of color correspond to greater changes in Ca^2+^ activity relative to baseline. (**E–F**) Cumulative distribution function plotting the z-score frequency by epoch in (**E**) male and (**F**) female subjects (KS test: By Sex-Before [D(117) = 0.3466, *P* value = 0.0014], Heat Start [D(117) = 0.3850, *P* value = 0.0002], Heat End [D(117) = 0.1815, *P* value = 0.2759], After [D(117) = 0.2611, *P* value = 0.0334]; Comparing each epoch within males results in significance with Heat Start and Heat End over Before and After [Before vs. Heat Start, D(98) = 0.4694, *P* value = 2.183e−05; Before vs. Heat End, D(98) = 0.4286, *P* value = 0.0001; Heat Start vs. After, D(98) = 0.4082, *P* value = 0.0003; Heat End vs. After, D(98) = 0.3265, *P* value = 0.0079], whereas comparing each epoch within females results in significance with Heat End over Before, Heat Start, and After [Before vs. Heat Start, D(136) = 0.2647, *P* value = 0.0135; Before vs. Heat End, D(136) = 0.4558, *P* value = 7.372e−07; Heat Start vs. Heat End, D(136) = 0.2352, *P* value = 0.0386; Heat Start vs. After, D(136) = 0.2941, *P* value = 0.0041; Heat End vs. After, D(136) = 0.3970, *P* value = 2.634e−05]). (**G**) Comparing average z-scores of BNST^CRF^ neurons by epoch for male and female subjects (Two-way mixed-model ANOVA with Tukey’s post hoc: Sex × Epoch interaction [F(3, 345) = 5.996, *P* = 0.0005], main effect of Sex [F(1, 115) = 13.20, *P* = 0.0004] and Epoch [F(2.153, 247.7) = 26.31, *P* < 0.0001]). denotes comparison of epochs for each sex, ^ denotes comparison of the same epoch between sexes. (**H**) Neuronal coactivity as measured by mean proportion of active BNST^CRF^ neurons. At each frame (0.2 s), the fraction of cells exhibiting positive z-scores was calculated and binned by epoch to reveal the proportion of concurrent BNST^CRF^ activity at phases relative to heat exposure (Two-way mixed-model ANOVA with Sidak’s post hoc: no Sex × Epoch interaction [F(3, 18) = 0.5414, *P* = 0.6601] or main effect of Sex [F(1, 6) = 0.9203, *P* = 0.3744]; main effect of Epoch [F(1.840, 11.04) = 4.163, *P* = 0.0475]). Data are shown as mean ± SEM. *P < 0.05; **P < 0.01; ***P < 0.001, ****P < 0.0001.

**Figure 3:**
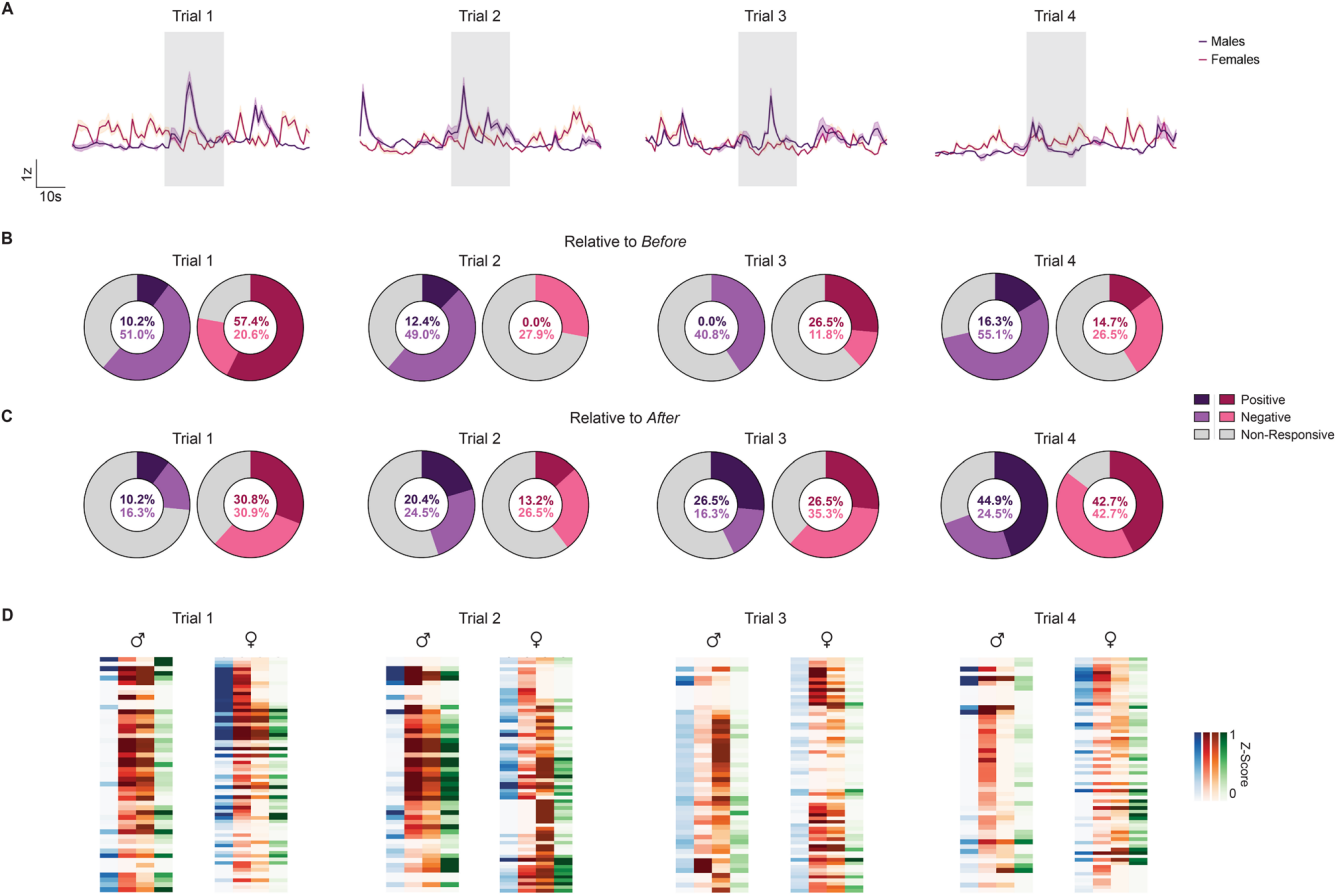
Progression of BNST^CRF^ Activity Across Noxious Heat Exposure Trials. (**A**) Average BNST^CRF^ traces of male (purple; n = 49 cells / 7–30 cells per mouse / 3 mice total) and female (magenta; n = 68 cells / 8–19 cells per mouse / 5 mice total) subjects by trial. Within each representative time window, the maximum heat duration (20 s) is indicated in the transparent blue block. (**B-C**) Percentage of pain responsive BNST^CRF^ neurons for male (purple) and female (magenta) subjects were determined relative to epochs surrounding the onset of (**B**) heat exposure [“Relative to *Before*”] and (**C**) paw withdrawal [“Relative to *After*”] in trials 1–4. Positive, negative, and non-responsive cells are indicated by darker purple/magenta, lighter purple/magenta, and grey respectively (Wilcoxon rank-sum, *P* < 0.05). Comparisons by trial and sex relative to the epochs surrounding heat onset (Two-way mixed-model ANOVA with Sidak’s post hoc: no Trial × Sex interaction [F(3, 18) = 0.0466, *P* = 0.9862] or main effect of Trial [F(1.092, 6.553) = 0.4473, *P* = 0.5435] and Sex [F(1, 6) = 1.510, *P* = 0.2651]) and paw withdrawal (Two-way mixed-model ANOVA with Sidak’s post hoc: no Trial × Sex interaction [F(3, 18) = 0.3670, *P* = 0.7777] or main effect of Trial [F(2.740, 16.44) = 0.3368, *P* = 0.7818] and Sex [F(1, 6) = 0.0008, *P* = 0.9776]) were not statistically significant. (**D**) Heatmap displaying the average z-score of individual BNST^CRF^ neurons by epoch for male and female subjects in trials 1–4. Epochs are color-coded: *Before* (blue), *Heat Start* (red), *Heat End* (orange), and *After* (green), with darker shades of color corresponding to greater changes in Ca^2+^ activity. Data are shown as mean ± SEM. *P < 0.05; **P < 0.01; ***P < 0.001, ****P < 0.0001.

**Figure 4:**
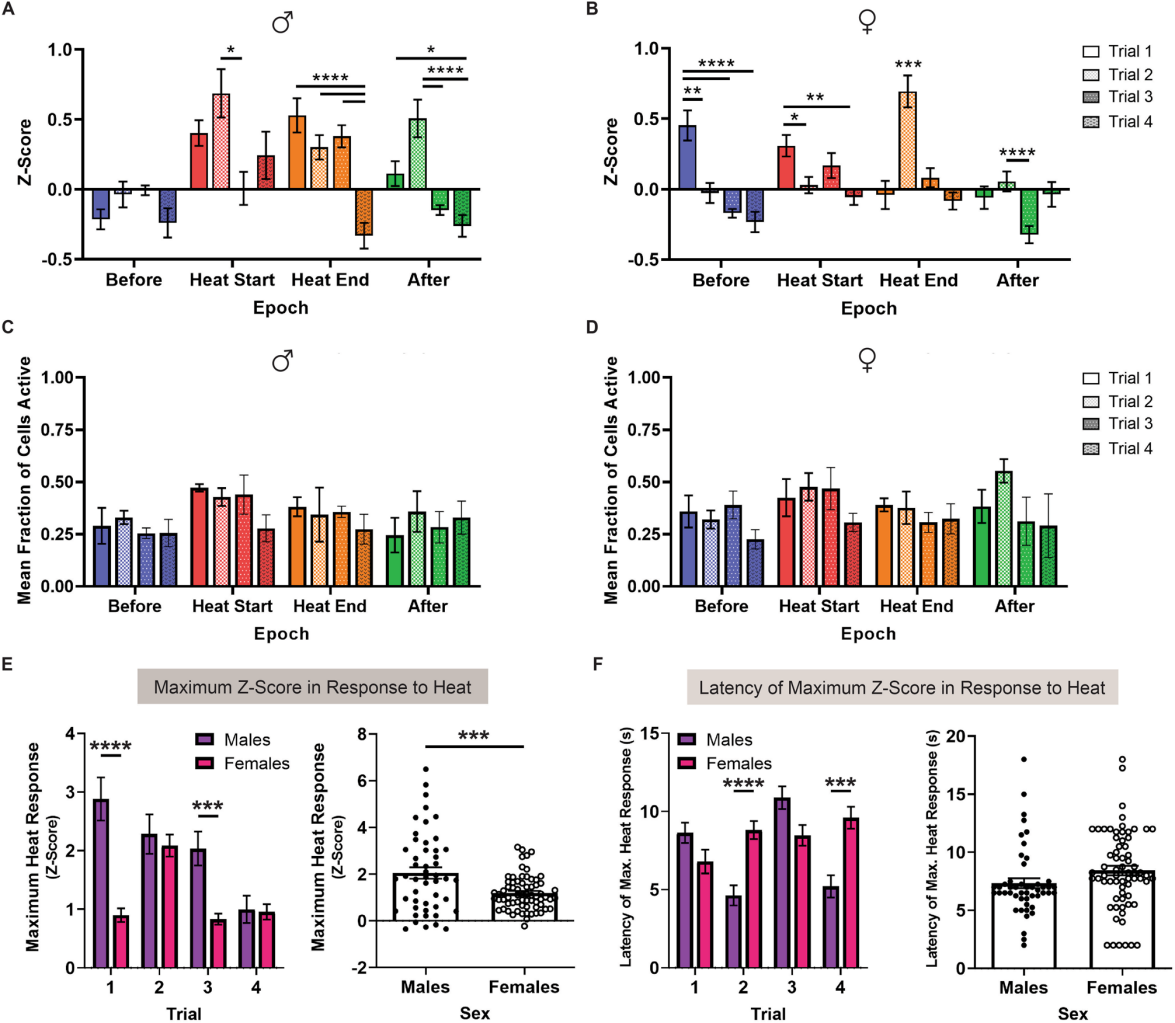
BNST^CRF^ Activity Changes with Repeated Noxious Heat Exposure. **(A–B**) Average z-score of BNST^CRF^ neurons by epoch for trials 1–4 in (**A**) male and (**B**) female subjects. Epochs are color-coded: *Before* (blue), *Heat Start* (red), *Heat End* (orange), and *After* (green) (Males = Two-way mixed-model ANOVA with Sidak’s post hoc: Epoch × Trial interaction [F(4.352, 208.9) = 8.148, *P* < 0.0001], main effect of Epoch [F(2.375, 114.0) = 16.66, *P* < 0.0001] and Trial [F(2.822, 135.4) = 10.63, *P* < 0.0001]; Females = Two-way mixed-model ANOVA with Sidak’s post hoc: Epoch × Trial interaction [F(3.150, 211.0) = 21.05, *P* < 0.0001], main effect of Epoch [F(1.679, 112.5) = 8.880, *P* = 0.0006] and Trial [F(2.491, 166.9) = 6.030, *P* = 0.0014]). (**C–D**) Average neuronal coactivity of BNST^CRF^ neurons by epoch for trials 1–4 in (**C**) male and (**D**) female subjects. Epochs are color-coded with the same system as (**A**–**B**): *Before* (blue), *Heat Start* (red), *Heat End* (orange), and *After* (green) (Males = Two-way mixed-model ANOVA with Sidak’s post hoc: no Epoch × Trial interaction [F(1.526, 3.052) = 1.046, *P* = 0.4252] or main effect of Epoch [F(1.419, 2.838) = 3.149, *P* = 0.1859] and Trial [F(1.178, 2.355) = 0.5741, *P* = 0.5447]; Females = Two-way mixed-model ANOVA with Sidak’s post hoc: no Epoch × Trial interaction [F(2.082, 8.329) = 0.9274, *P* = 0.4366] or main effect of Epoch [F(1.745, 6.981) = 2.102, *P* = 0.1935] and Trial [F(1.581, 6.326) = 1.041, *P* = 0.3871]). (**E**) (Left) Maximum z-score response to heat exposure by trial in male (purple) and female (magenta) subjects (Two-way mixed-model ANOVA with Sidak’s post hoc: Trial × Sex interaction [F(3, 345) = 14.96, *P* < 0.0001], main effect of Trial [F(2.609, 300) = 20.28, *P* < 0.0001] and Sex [F(1, 115) = 13.69, *P* = 0.0003]). (Right) Average maximum z-score response to heat in each trial by sex (unpaired t-test: t(115) = 3.70, *P* = 0.0003). (**F**) (Left) Latency of maximum z-score response to heat exposure by trial in male (purple) and female (magenta) subjects (Two-way mixed-model ANOVA with Sidak’s post hoc: Trial × Sex interaction [F(3, 345) = 16.90, *P* < 0.0001], main effect of Trial [F(2.717, 312.5) = 7.890, *P* < 0.0001], no main effect of Sex [F(1, 115) = 3.20, *P* = 0.0763]). (Right) Average latency of maximum z-score response to heat stimulus in each trial by sex (unpaired t-test: t(115) = 1.789, *P* = 0.0763). Data are shown as mean ± SEM. *P < 0.05; **P < 0.01; ***P < 0.001, ****P < 0.0001.

**Figure 5:**
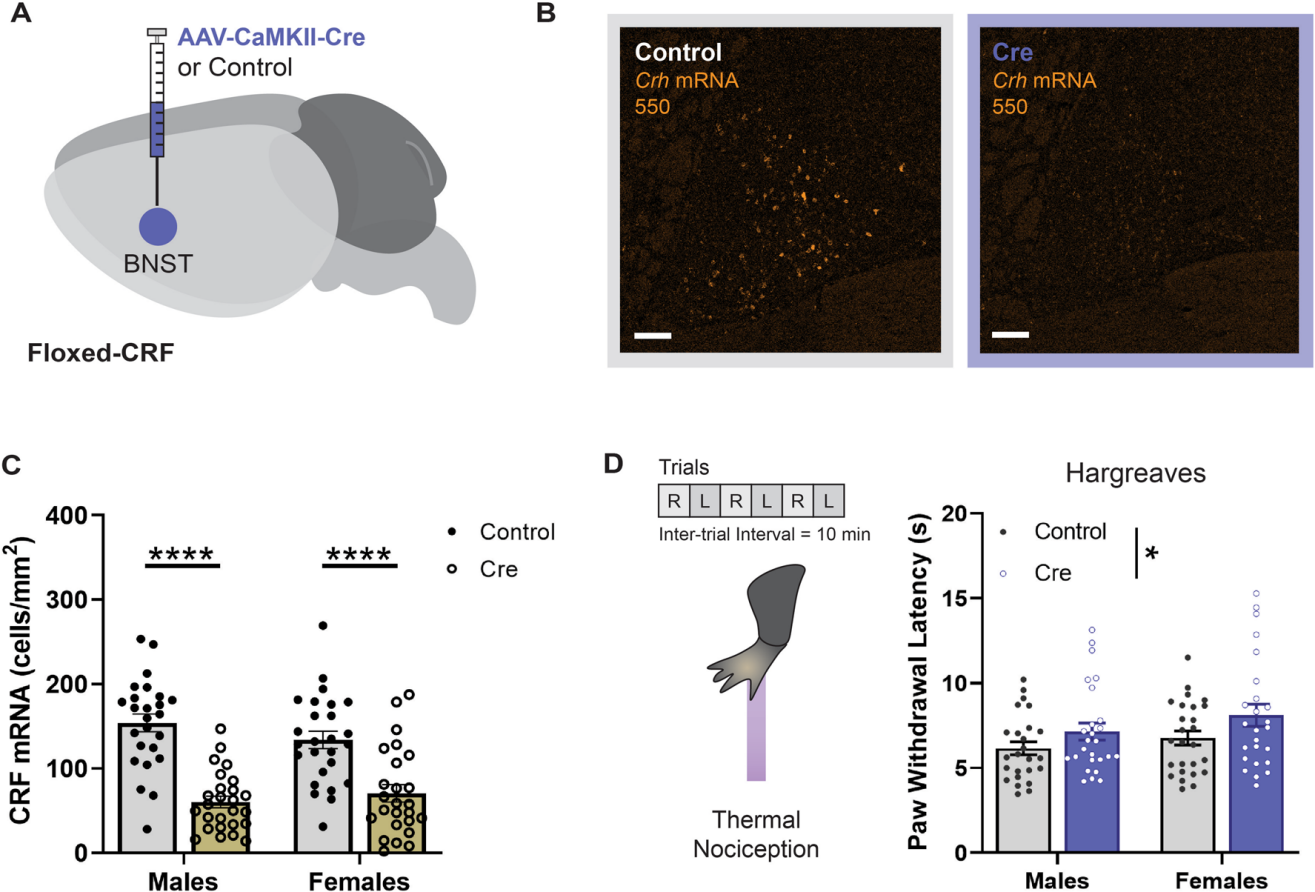
CRF Deletion in BNST Reduces Thermal Nociceptive Sensitivity. (**A**) Diagram of Cre-dependent approach for CRF deletion in BNST. (**B**) Representative histology of CRF mRNA expression in BNST of Floxed-CRF mice following virus infusion. (**C**) Quantification of CRF mRNA expression in BNST of male (n = 25–26) and female (n = 25–26) subjects by cells/mm^2^ (Two-way ANOVA with Tukey’s post hoc: no Sex × Virus interaction [F(1, 98) = 2.548, *P* = 0.1136] or main effect of Sex [F(1, 98) = 0.2667, *P* = 0.6067]; main effect of Virus [F(1, 98) = 66.74, *P* < 0.0001]). (**D**) (Left) Schematic of the Hargreaves test. Each paw was assessed using three replicate exposures, alternating between the right (R) and left (L) limb, resulting in six trials total. (Right) Thermal nociceptive sensitivity of male (n = 25–26) and female (n = 25–26) subjects (Two-way ANOVA with Tukey’s post hoc: no Sex × Virus interaction [F(1, 98) = 0.1253, *P* = 0.7242] or main effect of Sex [F(1, 98) = 2.471, *P* = 0.1192]; main effect of Virus [F(1, 98) = 5.388, *P* = 0.0223]). Data are shown as mean ± SEM. *P < 0.05; **P < 0.01; ***P < 0.001, ****P < 0.0001.

**Figure 6:**
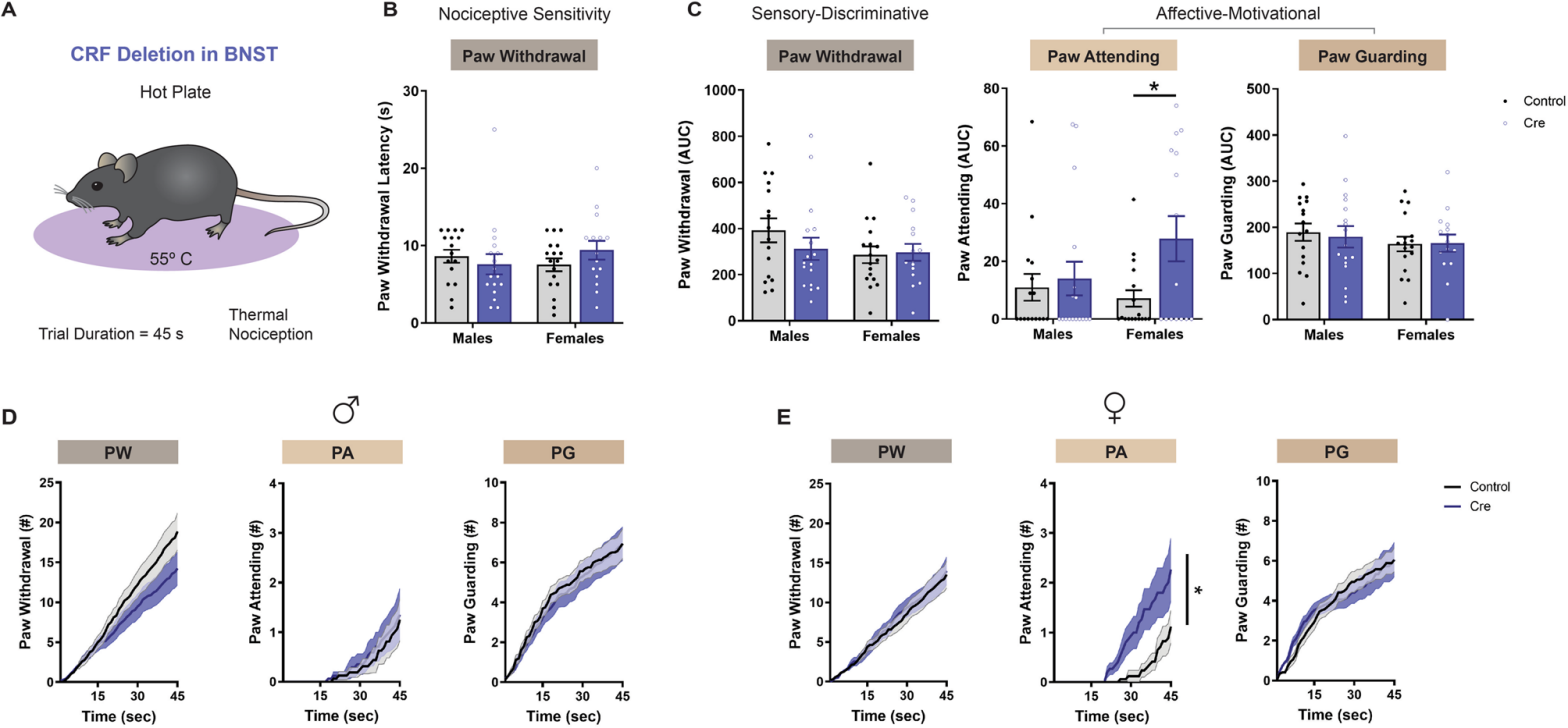
CRF Deletion in BNST Selectively Alters Sensory-Discriminative and Affective-Motivational Behaviors. (**A**) Schematic of hot plate test. (**B**) Thermal nociceptive sensitivity of male (n = 16–17) and female (n = 15–17) Floxed-CRF mice, as defined by latency to first paw withdrawal for each mouse (Two-way ANOVA with Tukey’s post hoc: no Sex × Virus interaction [F(1, 61) = 1.853, *P* = 0.1785] or main effect of Sex [F(1, 61) = 0.1124, *P* = 0.7386] and Virus [F(1, 61) = 0.1524, *P* = 0.6976]). (**C**) Quantification of sensory-discriminative and affective-motivational behaviors throughout the 45-s exposure to hot plate. Averaged area under the curve (AUC) of paw withdrawal (Two-way ANOVA with Tukey’s post hoc: no Sex × Virus interaction [F(1, 61) = 1.072, *P* = 0.3047] or main effect of Sex [F(1, 61) = 1.885, *P* = 0.1747] and Virus [F(1, 61) = 0.6208, *P* = 0.4338]), paw attending (Two-way ANOVA with Tukey’s post hoc: no Sex × Virus interaction [F(1, 61) = 2.605, *P* = 0.1117] or main effect of Sex [F(1, 61) = 0.8298, *P* = 0.3659]; main effect of Virus [F(1, 61) = 4.695, *P* = 0.0342]), and paw guarding (Two-way ANOVA with Tukey’s post hoc: no Sex × Virus interaction [F(1, 61) = 0.09333, *P* = 0.7610] or main effect of Sex [F(1, 61) = 1.026, *P* = 0.3152] and Virus [F(1, 61) = 0.04448, *P* = 0.8337]) are shown, with corresponding cumulative distribution functions displayed in (**D–E**). (**D–E**) Sensory-discriminative and affective-motivational behaviors are exhibited across time with cumulative distribution functions for (**D**) male and (**E**) female subjects (paw withdrawal: CON (M): 391.9 [341.0–442.9], CRE (M): 311.7 [263.2–360.2], CON (F): 285.9 [249.1–322.7], CRE (F): 296.8 [261.1–332.5]; paw attending: CON (M): 11.00 [4.672–17.33], CRE (M): 14.03 [6.087–21.97], CON (F): 7.147 [2.442–11.85], CRE (F): 27.87 [17.95–37.78]; paw guarding: CON (M): 189.3 [171.7–207.0], CRE (M): 179.3 [156.7–201.9], CON (F): 163.8 [147.6–180.0], CRE (F): 165.6 [147.8–183.4]). Data are shown as mean ± SEM. *P < 0.05; **P < 0.01; ***P < 0.001, ****P < 0.0001.
- Two-way repeated measures ANOVA with Tukey’s post hoc used for Fig. S2A,B.
- Two-way mixed-model ANOVA with Tukey’s post hoc used for Fig. 2G, S2C–F.
- Two-way mixed-model ANOVA with Sidak’s post hoc used for Figs. 2H, 3B,C, 4A–F, S1B–C.

Subjects were placed in Plexiglas boxes (4 × 4″ × 4″) on an elevated glass surface and habituated to the behavioral apparatus for 30–60 min to reduce novelty-induced locomotion and other factors that may confound nociceptive sensitivity measures. The mid-plantar surface of each hind paw was exposed to a series of noxious heat trials that sequentially alternated between left and right paws. After each heat exposure, a 10-min inter-trial interval was provided before the next trial. Six trials of heat exposure were conducted for all experiments except those using in vivo calcium imaging, which we restricted to 4 trials to prevent photobleaching. For Figs. 1, 2, 3, 4 (BNST^CRF^ imaging experiments), beam intensity was set to 15 on the IITC Plantar Analgesia Meter (IITC Life Science, Woodland Hills, CA), producing average basal paw withdrawal latencies of approximately 10 s in CRF-Cre mice. For Figs. 5, 6 (*Crf* deletion experiments), beam intensity was set to 25 on the IITC Plantar Analgesia Meter, producing average basal paw withdrawal latencies of approximately 6 s in Floxed-CRF mice. Cutoff times of 20 s were set for each trial to prevent tissue damage.

#### Von frey

Subjects were held in Plexiglas boxes on a custom-made elevated metal wire surface (90 × 20 × 30 cm) and habituated to the behavioral apparatus for 30–60 min to ensure stable readouts of nociceptive sensitivity. Nylon monofilaments of forces ranging from 0.008 to 2 g were applied to the hind paw using the simplified up-down method (SUDO)^8^. Starting with a mid-range force (0.16 g), the filament was applied to the mid-plantar surface of the hind paw for ten trials, then repeated with ascending or descending forces depending on the number of paw withdrawals. Withdrawal thresholds were defined as the minimum force filament that elicits a withdrawal reflex for ≥ 50% of the trials.

#### Tail immersion

Following a 30-min habituation to the behavioral room, subjects were restrained in Wypall fold wipers (Kimberly-Clark, Irving, TX) and tails were exposed to 50º C water in the test apparatus (Isotemp 110 Water Bath; Fisher Scientific, Hampton, NH). The tail flick latency was measured in two consecutive trials, where readings were taken 1 cm apart on the tail. The two latencies were then averaged together to indicate the reflexive nociceptive threshold of each subject. A cutoff time of 10 s was set to minimize tissue damage.

#### Hot plate

Subjects were positioned in a custom-made cylindrical Plexiglas container on an IITC Hot Plate Analgesia Meter (IITC Life Science, Woodland Hills, CA). To elicit sensory-discriminative and affective-motivational behaviors associated with pain, subjects were exposed to a 55º C black anodized aluminum plate (11″ × 10.5″ × ¾”) for 45 s. This sustained version of the hot plate test extends the traditional cutoff time of 20 s to 45 s to improve readout for behaviors beyond latency to the first paw withdrawal. Behaviors like paw withdrawal (rapid flicking of the limb), paw attending (licking of the limb), and paw guarding (intentional lifting of the limb for protection) reflect the extent to which subjects will engage in reflexive responses versus motivated behaviors to reduce the aversive aspects of pain^13^. Computer-integrated video cameras were used to record each session, allowing for blinded manual scoring of pain-related behaviors elicited by hot plate exposure. The behavioral apparatus was generously provided by the UNC Mouse Behavioral Phenotyping Laboratory.

#### Open field

Subjects were given 30 min to habituate to the behavioral room before being placed into a white Plexiglas open field (50 × 50 × 25 cm), where they could freely explore the arena for 10 min. The center of the open field was defined as the central 25% of the arena, where light levels were approximately 30 lx. Tracking of subject location and activity was collected with EthoVision (Noldus Information Technologies, Wageningen, Netherlands).

#### Elevated plus maze

Following a 30-min habituation period in the behavioral room, subjects were placed into the center of an elevated plus maze (EPM) and allowed to freely explore the arena for 10 min. During testing, subjects were able to explore two open arms (75 × 7 cm) and two closed arms (75 × 7 × 25 cm) that were bounded by a central area (7 × 7 × 25 cm). Light levels were set to approximately 15 lx to promote avoidance of exposed compartments in the apparatus, as measured by the relative time spent in the open and closed arms. Data related to subject location and activity in the EPM were collected with EthoVision (Noldus Information Technologies, Wageningen, Netherlands).

For all experiments, researchers were blinded to genotype/virus treatment.

### Ca^2+^ imaging with miniature microscope

#### Surgery

In CRF-Cre mice, a custom-prepared AAVDJ-EF1a-DIO-GCaMP6s virus (UNC Vector Core) was administered unilaterally in the dorsal region of BNST (250 nl in right hemisphere; relative to bregma: ML ± 0.90 mm, AP + 0.23 mm, DV − 4.35 mm). GRIN lenses (0.6 mm diameter/7.3 mm length; Inscopix, Palo Alto, CA) were implanted approximately 200 µm over the virus injection site (relative to bregma: ML ± 0.90 mm, AP 0.23 mm, DV − 4.15 mm) and secured with a dental cement headcap. To promote a stable recovery following this extensive surgical procedure, subjects were given daily injections of meloxicam (5 mg/kg, s.c.) for four consecutive days. Approximately four weeks after the initial surgery and recovery, baseplates (V2; Inscopix, Palo Alto, CA) were placed above the GRIN lens to stabilize the attachment of the miniature microscope. The baseplate attachment was performed in a visually guided manner, where the miniature microscope was incrementally lowered towards the implanted GRIN lens to identify the optimal distance needed to achieve a field-of-view (FOV) with the maximum number of fluorescent cells. The baseplate was then incorporated into the existing headcap with additional dental cement. Finally, baseplate covers were secured to the top of the headcap in order to protect the miniature microscope attachment site from environmental contaminants that may obscure the imaging FOV.

#### Behavior

Subjects were tested in the Hargreaves assay 6–8 weeks after the initial surgical procedure and 2–4 weeks after the baseplate procedure. Prior to testing, each subject was anesthetized with isoflurane (1–3%) in oxygen (1–2 l/min) in order to attach the miniature microscope to the baseplate. After two consecutive days of habituation to the miniature microscope, subjects were given a 15-min tethering session in their respective home cages and a 30-min tethering session in the Hargreaves apparatus. At the end of the tethering session, a 5-min imaging session for baseline calcium transients was recorded.

During testing, the mid-plantar surface of each hind paw was exposed to a series of noxious heat trials with 10-min inter-trial intervals. Each subject was exposed to 4 trials of heat (I15 on the IITC Plantar Analgesia Meter [IITC Life Science, Woodland Hills, CA] respectively), with radiant heat exposures sequentially alternating between left (contralateral) and right (ipsilateral) paws in the following order: trial 1: left; trial 2: right, trial 3: left; trial 4: right. For each trial, calcium transients were recorded with a 1-min window prior to onset of heat and a 1-min window following the offset of heat. Specific paw withdrawal latencies for each subject were used instead of a fixed duration to accurately capture individual nociceptive thresholds. Cutoff times of 20 s were set to prevent tissue damage.

All recordings of behavior in the Hargreaves assay were performed with Media Recorder and a computer-integrated video camera (#PC212XP, Miniature CCD Camera Series). Simultaneous recordings of BNST^CRF^ calcium transients were acquired using the nVista data acquisition software (Inscopix, Palo Alto, CA) at 20 frames per second (fps; Exp Time: 50 ms, Gain: 4–7, Ex. LED Power: 1).

#### Preprocessing

Using the Inscopix Data Processing Software (IDPS; Inscopix, Palo Alto, CA), recordings were cropped, temporally downsampled to 5 fps, and motion corrected. ΔF/F was then acquired to normalize each pixel value in the recording to a baseline value (as determined by mean frame). To identify temporally and spatially unique regions of interest (ROIs), we used PCA/ICA to detect putative cell bodies in the imaging FOV. After manually removing ROIs that did not correspond to cells, traces and events were exported as CSV files. Preprocessed ΔF/F recordings were further transported into maximum intensity projection images and movie files for representative depictions of the data.

#### Data analysis

Programmatic analysis was applied to extracted traces from IDPS using custom-written code in Python. Briefly, we transformed ΔF/F of baseline and test traces into z-scores for each subject using the formula: *Z* = *x—µ /* $\delta$, where the number of standard deviations ($\delta )$ that raw values (*x*) diverge from baseline values (*µ*) is determined. We then separated traces by epoch (*Before*, *Heat Start*, *Heat End*, *After*) for each subject and trail to determine averages by sex. Epoch timing was determined by dividing the duration of heat exposure into halves, so that each time block represented an equal amount of time for specific trials and subjects: *Epoch Length* = *[PWL / 2]*. Analysis was then performed for various combinations of data dimensionality based on these epochs.

We specifically compared the activity of individual male and female BNST^CRF^ neurons for the following metrics:

- *Average Z-Score by Epoch*: Mean z-score of epochs averaged across trials.
- *Average Z-Score by Trial and Epoch*: Mean z-score of epochs for each trial.
- Average *Neuronal Coactivity by Epoch*: Mean proportion of coactive cells by epoch. The fraction of active cells (# of cells with z-score > 0 / total # cells at frame x) was calculated for each frame (5 fps; 200 ms intervals) in the *Before*, *Heat Start*, *Heat End*, and *After* epochs. The coactive fraction of each individual subject and epoch was then averaged by sex for comparison.
- Average *Neuronal Coactivity by Trial and Epoch*: Mean proportion of coactive cells by epoch for each trial.
- *Cumulative Distribution Function Across Trials by Epoch*: Distribution of z-scores averaged across trials by binning the frequency of activity for each epoch.
- *Percent Responsiveness Across Trials*: Determine the proportion of cells that exhibit a statistically significant change in z-scores with heat exposure by using a Wilcoxon rank-sum test for each neuron × 4 trials and determining the fraction of cells exhibiting a prominent z-score change. Two notable events define comparisons of heat responsive neurons with the activity in surrounding epochs: “Heat Onset” (i.e. *Heat On* [*Heat Start* + *Heat End*] vs. *Before*) and “Paw Withdrawal” (i.e. *Heat On* vs. *After*). Positive differences in z-score correspond to activation in response to noxious heat exposure relative to surrounding epochs, while negative differences indicate a relative reduction in activity. Percentage of responsive cells was calculated by factoring in the activity changes of every neuron in all 4 trials.
- *Percent Responsiveness by Trials*: Determine the change between average z-scores of *Heat On* (i.e. *Heat Start* + *Heat End*) and surrounding epochs [vs. *Before*; vs. *After*] for each trial, with positive differences corresponding to activation in response to noxious heat exposure, and negative differences corresponding to inactivation. Percentage of responsive cells was calculated by taking the proportion of neurons exhibiting statistically different *Heat On* activity compared to *Before* and *After* in individual subjects and averaging these values for each trial.
- *Spike Magnitude by Trials*: Identify size of maximum value in *Heat On* (i.e. *Heat Start* and *Heat End*) epoch for each trial.
- *Spike Latency by Trials*: Identify time stamps for maximum value in *Heat On* (i.e. *Heat Start* and *Heat End*) epoch for each trial.

More information on the Python scripts used for analysis can be requested by contacting thomas_kash@med.unc.edu.

### In situ hybridization

Following isoflurane anesthetization and rapid decapitation, the brains of Floxed-CRF mice were collected and placed on aluminum foil, where they were immediately frozen on dry ice and stored in a − 80 °C freezer. Using a Leica CM3050 S cryostat (Leica Microsystems, Wetzlar, Germany), coronal sections of BNST (12 µm thickness) were obtained and directly mounted onto Superfrost Plus slides (Fisher Scientific, Hampton, NH), then kept at − 80 °C. In order to fluorescently label *Crf* mRNA in the BNST, slices were preprocessed with 4% PFA and protease reagent, incubated with target probes for mouse *Crh* (#316,091, RNAscope Probe-Mm-Crh; Advanced Cell Diagnostics, Newark, CA), then fluorescently labeled with a probe targeting the corresponding channel for the peptide (*Crh* in 550; Advanced Cell Diagnostics, Newark, CA). The processed slides were then covered using Vecta-Shield Mounting Medium with DAPI in preparation for imaging.

### Confocal microscopy

All fluorescent images were acquired with the Zeiss 800 Upright confocal microscope and ZenBlue software (Carl Zeiss AG, Oberkochen, Germany), with equipment access granted through the Hooker Imaging Core at UNC Chapel Hill. Validation of virus expression/injection site, GRIN lens placement, and immunoreactivity were accomplished with tiled and serial z-stack images obtained through a 20 × objective (3 × 3 tile, 2 μm optical slice thickness, 12 µm total thickness). Images were processed in FIJI^69^ for manual counting and ZenBlue (Carl Zeiss AG, Oberkochen, Germany) for area measurements in each ROI. Inclusion criteria for subjects was based on localizing injections and/or implants to the anterior portion of the dorsal BNST (relative to bregma: ML ± 0.90 mm, AP + 0.23 mm, DV − 4.35 mm). For imaging experiments, we verified the presence of (i) cortical damage indicating lens placement, (ii) GCaMP restricted to the BNST, and (iii) similar placement of components in the targeted anterior/posterior range of the BNST. For deletion experiments, we confirmed genetic knockdown by calculating the number of BNST neurons expressing *Crh* mRNA puncta per mm^2^ in the same anterior and dorsal positioning described above. All subjects included in the paper were able to meet these criteria.

### Statistical analysis

Single-variable comparisons were made using unpaired t-tests. Group comparisons were made using two-way ANOVA, two-way ANCOVA, two-way repeated measures ANOVA, or two-way mixed-model ANOVA depending on the number of independent and within-subjects variables in a data set. Following significant interactions or main effects, post-hoc pairwise t-tests were performed and corrected using Sidak’s or Tukey’s post-hoc tests to control for multiple comparisons. Results of statistical testing are reported in figure legends with significance indicated through markers on figures. Data are expressed as mean ± standard error of the mean (SEM), with significance for *p* values below 0.05 (*P < 0.05, **P < 0.01, ***P < 0.001, ****P < 0.0001). All data were analyzed and visualized with standard statistical software packages from GraphPad Prism 8 (GraphPad Software, San Diego, CA) and SciPy^80^.

Details on the statistical tests used:

- Unpaired t-test used for Figs. 4E–F, S1A.
- Wilcoxon rank-sum test used for Figs. 1D, 3B,C.
- Kolmogorov–Smirnov test used for Fig. 2E,F.
- Area under the curve used for Fig. 6D,E.
- Simple linear regression used for Fig. S4A–F, Table 1.
- Two-way ANCOVA used for Fig. S1A–S4F, Table 1.
- Two-way ANOVA with Tukey’s post hoc used for Figs. 1B,C, 5C,D, 6B,C, S3A–H.

## Results

### BNST^CRF^ neurons are endogenously recruited by noxious heat

To assess how BNST^CRF^ neurons process the experience of pain, we used a head-mounted miniature microscope to track in vivo somatic calcium activity of individual *Crh* neurons in the BNST during hind paw exposure to noxious heat (Fig. 1). By implanting a GRIN lens and injecting an adeno-associated virus carrying Cre-inducible GCaMP6s (AAVDJ-EF1a-DIO-GCaMP6s) in the right hemisphere BNST of adult male (n = 3 mice; 49 cells; 7–30 cells per mouse) and female (n = 5 mice; 68 cells; 8–19 cells per mouse) CRF-Cre mice (Fig. 1A), we were able to continuously monitor BNST^CRF^ activity during the Hargreaves test, a standard measure of thermal nociceptive sensitivity consisting of repeated trials of noxious heat exposure. We specifically focused on BNST^CRF^ activity surrounding the onset and offset of heat, two timepoints that represent the initial stimulus detection and paw withdrawal response respectively (Fig. 1B,C). Since the length of heat exposure was determined by paw withdrawal latencies (PWL), a measure that varies by subject and trial (Fig. S1), we analyzed activity using epochs relative to heat exposure: *Before*, *Heat Start*, *Heat End*, and *After* (Fig. 2A–D; [Epoch Length = PWL / 2]; see “*Ca*^*2*+^ *imaging with miniature microscope*” section in Methods for details). Comparisons of averaged BNST^CRF^ activity across four trials of heat exposure revealed sex-specific recruitment by epoch, with higher fold calcium changes observed for *Heat Start* and *Heat End* in males and *Heat End* in females (Fig. 2E–G; main effect of Epoch: F_2.153, 247.7_ = 26.31, *P* < 0.0001). Average z-scores of *Before* and *Heat Start* epochs differed by sex, with males showing a larger magnitude of BNST^CRF^ activity in the earlier phases of stimulus exposure than females (Fig. 2G; main effect of Sex: F_1, 115_ = 13.20, *P* = 0.0004). These results show that BNST^CRF^ neurons respond to noxious heat with distinct levels of activation in male and female mice.

Although magnitude of response is a common marker for neuronal encoding, ensembles of activity have been posited to play a distinct role in how neurons transmit information^25^. To determine whether BNST^CRF^ neurons exhibit neuronal coactivity during exposure to noxious heat, we calculated the average fraction of simultaneously active (z-score > 0) cells in each frame of an epoch. Interestingly, coactivation of neurons also differed in the earlier phases of stimulus exposure, with females exhibiting increased BNST^CRF^ coactivity between the transition from *Before* to *Heat Start* (Fig. 2H; main effect of Epoch: F_1.840, 11.04_ = 4.163, *P* = 0.0475). However, comparable patterns of coactivity were not found in their male counterparts (Fig. 2H).

Notably, while there were sex differences in the magnitude and coactivity of responses when examining average z-scores, the proportion of cells showing statistically significant heat responses relative to surrounding epochs did not differ between male and female subjects (Fig. 1D). Comparisons of *Heat On* activity relative to surrounding epochs resulted in z-score changes for only 9.7–28.3% of cells with positive responses (i.e. *Heat On*-*[Before* or *After]* > 0 for cells showing a significant difference by epoch with a Wilcoxon rank-sum test) and 20.4–49.0% of cells with negative responses (i.e. *Heat On*-*[Before* or *After]* < 0 for cells showing a significant difference by epoch with a Wilcoxon rank-sum test) when averaged across trials (Fig. 1D). Categorizing z-scores by responsivity type for each cell further validated that this small percentage of positive responding neurons were the main drivers of BNST^CRF^ activity during exposures to noxious heat in both sexes (Fig. S2).

### BNST^CRF^ neurons exhibit dynamic activity with repeated exposure to noxious heat

We next evaluated BNST^CRF^ activity on a trial-by-trial basis to determine how neuronal responses to noxious heat are impacted by sequential nociceptive exposures (Fig. 3A). Higher magnitudes of z-scores were generally observed in the first two trials of *Heat Start* and *Heat End* epochs (Fig. 4A,B), while neuronal coactivity did not exhibit any differences between the initial and final exposures to heat (Fig. 4C,D). Additional trial effects were revealed when comparing maximal magnitude z-scores by sex. Although male mice exhibited BNST^CRF^ responses with greater maximal magnitudes of activation than females (Fig. 4E; t_115_ = 3.70, *P* = 0.0003), maximum heat responses diminished throughout the progression of trials for males (Fig. 4E; main effect of Sex: F_1, 115_ = 13.69, *P* = 0.0003), indicating that earlier heat exposures elicit greater magnitudes of BNST^CRF^ response in males. By contrast, the latency of maximum response became more delayed with additional trials for female mice (Fig. 4F; Trial × Sex interaction: F_3, 345_ = 16.90, *P* < 0.0001), resulting in a trend for later peak times following repeated heat exposure in females (Fig. 4F; t_115_ = 1.789, *P* = 0.0763).

Tracking the percentage of heat responsive BNST^CRF^ neurons across trials, we observed a combination of positive and negative changes in activity that varied in distribution with repeated exposures (Fig. 3B,C). However, the percentage of heat responsive BNST^CRF^ neurons for each trial did not significantly differ by trial progression or sex. These data suggest that multiple exposures to acute thermal nociception can drive distinct changes in the magnitude and timing of cell responses, but not the total proportion of responsive cells, for male and female mice.

Together, our imaging results demonstrate that BNST^CRF^ neurons are activated by noxious heat in a sex-specific manner, with male and female mice relying on divergent mechanisms to encode for nociceptive stimuli. Specifically, by exhibiting a greater magnitude of activation in males, more prominent coactivation in females, and significant alterations in activity with additional heat exposures, we show that BNST^CRF^ neurons are a dynamic source of encoding for the pain experience.

### Genetic impairment of CRF expression in BNST reduces pain sensitivity

Given the heterogenous nature of peptide expression in the BNST and a fundamental lack of insight on the conditions that drive CRF release, it was unclear whether the observed BNST^CRF^ responses to noxious heat in our imaging experiments would translate to a functional role for CRF in the modulation of pain-related behaviors. To address this issue, we genetically impaired CRF expression in the BNST by bilaterally injecting an adeno-associated virus carrying Cre (AAV5-CaMKIIa-Cre-GFP) or a control virus (AAV5-CaMKIIa-GFP) in the BNST of adult male and female Floxed-CRF mice (Fig. 5A). This approach resulted in an approximate 50–60% reduction in *Crh* mRNA-positive neurons in the BNST (Fig. 5B,C; main effect of Virus: F_1, 98_ = 66.74, *P* < 0.0001; see Fig. S4 and Table 1 for correlations between CRF expression in BNST and pain-related behaviors). Using the Hargreaves test, we found that CRF deletion reduced thermal nociceptive sensitivity compared to control mice (Fig. 5D; main effect of Virus: F_1, 98_ = 5.388, *P* = 0.0223). Notably, the extent of reduction for *Crh* mRNA expression and pain sensitivity did not differ between male and female subjects (Fig. 5C,D; no main effect of Sex for *Crh* mRNA [F_1, 98_ = 0.2667, *P* = 0.6067] or Hargreaves [F_1, 98_ = 2.471, *P* = 0.1192]). Identical sensitivity changes with CRF deletion were reported for the Von Frey test (Fig. S3A; main effect of Virus: F_1, 98_ = 10.76, *P* = 0.0014), but not the tail immersion test (Fig. S3B; no main effect of Virus: F_1, 98_ = 1.944, *P* = 0.1663), consistent with a supraspinal mechanism of pain modulation. These results show that a deficiency of CRF in the BNST generates increased pain thresholds, suggesting that the presence of CRF may be necessary to preserve the sensory components of pain for both male and female mice.

### Genetic impairment of CRF expression in BNST differentially impacts the sensory and affective-motivational components of pain

Considering that CRF signaling in the BNST plays an important role in multiple components of the pain experience^50,76^, we tested the same CRF deletion and control mice in an extended hot plate test^13^. Differing from a standard hot plate protocol that terminates after the first paw withdrawal or a relatively brief predetermined cut-off time, this prolonged form of the test lasts 45 s to promote the expression of both sensory-discriminative (e.g. paw withdrawal [rapid flicking of the limb]) and affective-motivational (e.g. paw attending [licking of the limb] and paw guarding [intentional lifting of the limb for protection] behaviors (Fig. 6A). Although genetic impairment of CRF expression in the BNST did not alter sensory-discriminative responses to the hot plate, as measured by paw withdrawal latency (Fig. 6B) and cumulative paw withdrawal behaviors (Fig. 6C), or affective-motivational responses such as paw guarding (Fig. 6C), there was a marked increase in paw attending for Cre-treated female mice (Fig. 6C–E; main effect of Virus: F_1, 61_ = 4.695, *P* = 0.0342). This sex-specific change in paw attending suggests a divergent role for CRF in the BNST and the sensory-discriminative/affective-motivational components of a prolonged, inescapable thermal nociceptive stimulus. Remarkably, reducing CRF expression in the BNST did not impact other pain-independent affective-motivational behaviors such as avoidance (Fig. S3D and G) and locomotor (Fig. S3E and H) behaviors in the open field (Fig. S3C) and elevated plus maze (Fig. S3F), indicating that the aversive contributions of CRF deletion in the BNST are specific to pain-related contexts like the extended hot plate.

## Discussion

Pain is a multi-faceted experience that necessitates adaptive coping strategies to mitigate harm. In the present study, we identified a subpopulation of BNST neurons that can respond to and modulate these pain-related behaviors through local expression of CRF. Utilizing genetically targeted manipulations of CRF-containing neurons and CRF itself in the BNST, we were able to demonstrate the dynamic recruitment of BNST^CRF^ activity by noxious heat, as well as the modulatory role of CRF on pain in male and female mice. Our results support and expand upon earlier work on intra-BNST CRF signaling and pain^50,76^, which has historically been limited to the role of CRF receptors in the BNST of male rodents, by uncovering previously unknown sex-specific contributions of local CRF production. These findings provide valuable insight on how nociceptive microcircuits in the BNST contribute to the experience of pain.

### BNST^CRF^ neurons dynamically respond to noxious heat across trials in a sex-specific manner

Our imaging experiments determined that BNST^CRF^ neurons generate somatic Ca^2+^ activity in the presence of noxious heat. Since the greatest increases in activity were observed at the *Heat Start* and *Heat End* epochs for males and the *Heat End* epoch for females, we conclude that BNST^CRF^ neurons are activated by noxious heat in both sexes. Sex differences at the *Before* and *Heat Start* epochs, where the initial detection of the stimulus elicited greater neuronal responses in males, additionally suggest that neuronal representations of pain differ by sex in the early phases of heat exposure. This difference in early phase processing may indicate a higher probability of detecting the nociceptive stimulus, since males exhibited trends for BNST^CRF^ neurons with faster maximum heat response times and greater magnitudes of activation. By contrast, females exhibited greater BNST^CRF^ coactivity changes in the early phases of heat exposure, with the most prominent increases in simultaneous activity happening between the transition from *Before* to *Heat Start*. We posit that these disparities in early phase activity reflect sex-specific mechanisms of nociceptive processing. Considering that salience is an important predictor of pain sensitivity and analgesic efficacy^9^, it is possible that alterations in the timing and synchrony of BNST^CRF^ responses will change nociceptive detection and contribute to pain sensitivity in a sex-specific manner. This is an intriguing possibility given that BNST neurons expressing *Crh* or the prepronociceptin gene have been reported to exhibit in vivo responses to motivationally salient and aversive stimuli such as predator odor^27,61^. These observations support prior conclusions that the BNST encodes for salient threats that may cause physical or emotional harm^2,9,29,50,73^ (Herrmann et al.^33^).

Since salience can either enhance or diminish the importance of a nociceptive signal with experience^9^, we additionally evaluated whether BNST^CRF^ responses to heat can change with repeated exposure. Comparisons by sex revealed increasingly lower magnitudes of activity for males and slower maximum heat response times for females as trials advanced. These progressive changes in magnitude and latency suggest that BNST^CRF^ neurons encode for novelty, a distinctive feature that contributes to the nociceptive impact of a stimulus, with the extent of response decreasing and becoming more delayed as subjects habituate to the experience. Clinical reports similarly show that repeated exposure to a nociceptive stimulus leads to dissociations in stimulus representation by the brain, with the BNST being implicated in the gating processes that determine the significance of environmental stimuli^7,33^. Reductions in BNST^CRF^ activity may thus reflect the diminished salience of pain as stimulus exposures accumulate. In contrast to related structures like the basolateral amygdala and the central nucleus of the amygdala [CeA], which exhibit neuronal responses that positively scale with increasing pain intensity and repeated nociception^19,28,38,82^ (for a non-responsive example, see Hua et al*.*^34^), BNST^CRF^ responses are distinctly desensitized with repeated pain exposures.

Despite these sex differences reported in the size and timing of responses across trials, it is important to recognize that these differences did not translate to test performance, as thermal nociceptive sensitivity was comparable between male and female mice across trials. This finding suggests that the observed discrepancies in BNST^CRF^ encoding between males and females are indicative of a sex-specific means to process pain rather than a mechanism to drive behavioral variation. Quantitative comparisons have shown that female subjects are more sensitive than males in 85.4% of rodent studies^53^, so our recordings of in vivo BNST^CRF^ responses to various components of the pain experience offer unique insight on how comparable nociceptive sensitivity can be processed in a sex-specific manner.

Similarly, the percentage of BNST^CRF^ neurons that respond to noxious heat did not vary by sex. However, we did observe variability in the types of BNST^CRF^ responses on a single cell level in both sexes, where responses to heat were comprised of both increasing and decreasing z-scores compared to activity in surrounding epochs. Previous data from our group has suggested that multiple populations of CRF neurons in the BNST differentially contribute to the regulation of affective behaviors^48^, with BNST^CRF^ neurons that form local inhibitory synapses playing a greater role in aversion. Assuming that the magnitude of response is a reliable metric of activation, these data suggest a model where heightened BNST^CRF^ activation is accompanied by increased inhibitory tone. Suppression of local GABAergic projection neurons by CRF has been shown to inhibit dopamine neurons in the ventral tegmental area^48,75^, so it is possible that the rapid decreases in BNST^CRF^ activity observed prior to paw withdrawal are providing a motivational signal to move away from the noxious stimulus. Although BNST^CRF^ neurons exhibit local inhibitory connections, it should be noted that they also have long-range connections with regions involved in motivated behavior, such as the lateral hypothalamus, where activation of BNST^CRF^ projections can induce conditioned place aversion^17,27^. These distinct CRF populations contributing to heterogeneous motivational behaviors in the BNST may explain how pain simultaneously promotes self-preservation while negatively influencing emotion. In future experiments, researchers should more rigorously examine the functional contributions of BNST^CRF^ subpopulations based on these varied responses to nociceptive stimuli, with special consideration for how the proportion of pain responsive neurons and magnitude/timing of activity interact to inform function.

An important caveat to consider with these results is the possibility that BNST^CRF^ neurons exhibit similar responses to a wide range of tactile stimuli, regardless of their nociceptive properties. Although explicit comparisons of BNST^CRF^ activity in the presence of nociceptive and non-nociceptive stimuli were not performed, our imaging experiments were designed to have a low intensity (“resting”) heat beam contact the hind paw to ensure accurate midplantar targeting prior to the onset of a higher intensity (“test”) heat beam. It is likely then that the *Before* epoch is already capturing BNST^CRF^ responses to a non-nociceptive stimulus. Under this interpretation, our data shows that the nociceptive (i.e. “test” heat beam) stimulus is driving greater BNST^CRF^ activity than the non-nociceptive (i.e. “resting” heat beam) stimulus. Follow-up studies that intend to compare the impact of nociceptive and non-nociceptive exposures in a more rigorous manner should incorporate concurrent trials exclusively comprising of “resting” heat beam exposures, which would allow for more analogous time-scale comparisons to the “test” heat beam exposures described in the present study. If BNST^CRF^ neurons are indeed encoding the salient aspects of nociception, performing a battery of exposures with a wider range of sensory impact (i.e. using various modalities of noxious stimuli [thermal vs. mechanical vs. inflammatory] that span the spectrum of nociceptive impact) could further refine our understanding of how this local CRF population encodes the experience of pain.

While our findings highlight the use of in vivo calcium dynamics in BNST^CRF^ neurons to understand pain, we acknowledge that the mechanisms contributing to sex-specific encoding remain to be determined. Recent evidence for sex-dependent excitatory transmission and synaptic facilitation in BNST^CRF^ neurons suggests that highly active glutamatergic synapses in female mice prevent increases in excitatory tone that males are otherwise susceptible to^47^. Such differences in intrinsic excitability have major implications on how we interpret relative changes in calcium transients, since we measure these outputs by (i) normalizing pixels within each field-of-view (ΔF/F) and (ii) comparing nociceptive responses to baseline activity. Having lower basal excitation may explain why BNST^CRF^ neurons can show higher magnitude responses to nociceptive stimuli in male mice, whereas higher basal excitation may promote a greater likelihood of simultaneous activity in female mice. Although further clarification is necessary, the conclusions established from our study and others may be important steps towards an integrated understanding of how the intrinsic cell properties of BNST^CRF^ neurons directly contribute to sex-specific in vivo responses to pain. Understanding how these BNST^CRF^ responses operate in the context of the greater BNST network will be key to decoding these outcomes in future studies.

### CRF in the BNST modulates the expression of sensory and affective-motivational components of pain

While previous studies have found that pharmacological manipulation of CRF signaling in the BNST can modulate multiple aspects of pain-related behavior^35,40,76,77^, the source of this CRF is unclear, as the BNST contains locally produced CRF and receives CRF input from the CeA^24,66^. In examining how local production of CRF can impact these pain-related behaviors, our work has revealed that both thermal and mechanical nociceptive sensitivity are impacted by impairing CRF expression in the BNST. These results are reminiscent of previously published effects showing that CRF_2R_ antagonism reduces mechanical and colonic nociception in stress-free conditions^76^. However, similar pharmacological manipulations failed to alter nociceptive behaviors associated with formalin and only blocked conditioned place aversion^35,40^. In the context of our results, these studies suggest that CRF signaling in the BNST selectively contributes to the sensory and affective-motivational components of pain based on the type of nociceptive stimulus and the context of presentation. As others have hypothesized, the duration of a nociceptive stimulus and the capacity of an animal to prevent further harm by the stimulus (i.e. escapability) may be important factors in pain processing^3,26,64^. If we categorize these known effects of CRF signaling in the BNST by phasic-acting/escapable nociceptive exposures like the Hargreaves and Von Frey tests versus tonic-acting/inescapable nociceptive exposures like formalin, we can posit that CRF is more likely to alter the sensory components of pain when challenged with shorter-lasting stimuli, while the affective-motivational components are more likely to be influenced by longer-lasting stimuli.

In support of these observations, we report that local CRF expression selectively modulates affective-motivational behaviors in the hot plate. Of the measures for the emotional aspects of pain, only paw attending was altered by CRF deletion, as females exhibited more licks to the site of injury in Cre-treated mice than controls. Unlike the Hargreaves and Von Frey tests, where exposure to the nociceptive stimulus terminates after a paw withdrawal or a fixed number of rapid prodding, the extended hot plate exposes subjects to heat for a duration where the thermal nociceptive stimulus is tonic-acting/inescapable compared to the standard version of the test. This change in the timing and intensity of nociceptive stimulus presentation may explain why CRF deletion can generate seemingly contradictory phenotypes like sex-independent pain relief and sex-dependent exacerbation of an adaptive coping behavior in the same group of mice. Discrepancies in these pain-related outcomes likely indicate a functional distinction in how CRF signaling in the BNST interacts with specific nociceptive stimuli as phasic- or tonic-acting stressors.

However, features like timing and intensity may not fully explain how CRF in the BNST contributes to pain. Contrary to the anti-nociceptive effects observed with acute nociceptive exposures in the Hargreaves and Von Frey tests, we found that CRF deletion in the BNST did not impact the sensory components of pain in the hot plate when measured by initial latency and cumulative number of paw withdrawals. Differences in the presentation of stimulus may explain this lack of contingency across assays. We specifically found that heat exposure to an individual hind paw in the Hargreaves test resulted in shorter-lasting PWLs than exposing all four paws to a higher temperature of heat in the hot plate. Diffuse exposures to thermal stimuli (i.e. noxious heat contacts all four paws) were thus more inclined to produce higher nociceptive thresholds than concentrated (i.e. noxious heat contacts an individual hind paw) exposures, regardless of temperature. Any influence of CRF expression was likely masked by this ceiling effect of PWLs in the hot plate. The conditional impact on thermal nociception reported here emphasizes the need for more thorough examinations on how contextual features of nociceptive exposures—such as timing, intensity, and diffusion/concentration—work together to influence pain. Information that more concretely indicates potential for harm is expected to have a greater impact to how CRF in the BNST modulates pain-related behaviors.

### The selective role of sex dependent intra-BNST CRF signaling in pain

The discrete interactions observed between nociception, context of presentation, and adaptive behavior could in part be explained by sex-dependent approaches to processing threats in the environment. As our imaging data suggest, the same nociceptive experience could require differential engagement of intra-BNST CRF signaling in male and female subjects. The BNST has been implicated in pain and salience through its role in hypervigilant threat monitoring, a form of sustained attention reserved for hostile stimuli^10,33,37^. Systemic administration of CRF impairs sustained attention, with the greatest impact found in the diestrus phase of the estrous cycle for female rodents^11^. Under the assumption that estrous cycle influence on sustained attention applies to discrete processing of tonic-acting/inescapable nociceptive stimuli between males and females, it is possible that impairing CRF expression in the BNST exclusively enhances the detection of longer-lasting pain in females to increase active coping behaviors. By contrast, the same CRF manipulation may be less susceptible to estrous influence when confronted with phasic-acting/escapable nociceptive stimuli, since the detection of acute pain may depend less on sustained attention^9,33,73^.

While the present study did not track fluctuations in the estrous cycle, it is an intriguing possibility for sex hormones to conditionally interact with CRF signaling based on salience, so that tonic-acting/inescapable contexts increase the likelihood of sex-specific responses to pain. This theoretical framework supports earlier work establishing sex differences in peptide distribution, receptor expression, and stress response as broad features of CRF signaling throughout the brain^5,78^, with many of these phenotypes being the product of hormonal surges during development^5^. Whether similar sex-specific functions of CRF signaling apply to the BNST was not addressed in previous evaluations of CRF signaling and pain, as these studies were performed exclusively in male subjects^23,35,40,76,77^. By consequence, the findings described in the present study are the first to compare how local production of CRF in the BNST contributes to different modalities of pain in both sexes. Additional studies on the impact of the estrous cycle on nociceptive stressors of varying duration/escapability may be necessary to more fully understand how CRF signaling in the BNST contributes to discrete pain processing and modulation in male and female mice.

### Null effects of CRF in the BNST on anxiety-like behaviors

Interestingly, no differences in avoidance or locomotors behaviors were observed with impaired CRF expression in the BNST. Previous studies have shown that CRF infusion in the BNST is anxiogenic and produces conditioned place aversion, while pharmacological inhibition of CRF_1R_ but not CRF_2R_ in the BNST is anxiolytic^65^. Furthermore, work from our lab has shown that chemogenetic inhibition of BNST^CRF^ neurons reduces avoidance behavior in the open field^59^. Our results demonstrate that genetic targeting of CRF production does not reduce avoidance behaviors, similar to the null effects observed in a previous study with CRF overexpression in the BNST^70^, suggesting that there may be other factors in BNST^CRF^ neurons driving these changes. Although there has yet to be a comprehensive investigation on BNST^CRF^ co-expression and its relative contributions to avoidance behaviors, distinct anxiogenic roles have been identified for GABA, CRF, and other modulatory factors in the CeA^60^. A similar segregation of anxiogenic contributions may exist in BNST^CRF^ neurons as well. It remains possible, however, that CRF plays a prominent role in avoidance behaviors, and that a greater reduction in *Crh* mRNA beyond 50–60% is necessary to produce anxiolytic effects. Alternatively, it is feasible that anxiety-like states are more robustly regulated by external CRF contributions than local contributions within the BNST.

### CRF in the BNST as a potential therapeutic target for chronic pain

Taken together, our work raises important questions about the relationship between the dynamic activity of BNST^CRF^ neurons and CRF release. Unlike classical neurotransmitters such as GABA, the kinetics of release and activity of neuropeptides can be much longer. Elegant work from the Minami group has demonstrated that CRF can be released in the BNST over the course of an hour following formalin injection^35^, so it is possible that some of the changes we observed over time in our in vivo imaging studies were due to local release of CRF. This is a particularly interesting possibility in the context of chronic pain, since long-term injury has been reported to drive functional upregulations of CRF signaling and putative CRF activity in the BNST^31,35,63,75^. Interestingly, treating adult male human subjects with CRF has been shown to relieve pain during persistent inflammatory states such as post-operative pain but lack analgesic properties for healthy individuals^32,46^, suggesting that lasting pain conditions can change how CRF modulates pain. Future studies examining the relationship between chronic pain and in vivo dynamics of BNST^CRF^ activity in response to noxious stimuli will therefore be essential for a comprehensive understanding of peptide function in pain processing and modulation.

To summarize, our findings have established a previously uncharacterized sex-specific role for CRF in the BNST and pain-related behaviors that will provide important insight into the aversive microcircuitry of the extended amygdala. We hope that this knowledge will present a viable path towards more equitable approaches to recognizing and managing pain for both sexes.

## Supplementary Information

Supplementary Information.
