# Supplementary material for: Corticotropin-releasing factor neurons in the bed nucleus of the stria terminalis exhibit sex-specific pain encoding in mice
Source: Sci Rep. 2021 Jun 14;11:12500. doi: 10.1038/s41598-021-91672-8 (PMC8203647; doi:10.1038/s41598-021-91672-8)
Supplement: Supplementary file 1 — Supplementary Information. [file 41598_2021_91672_MOESM1_ESM.pdf]

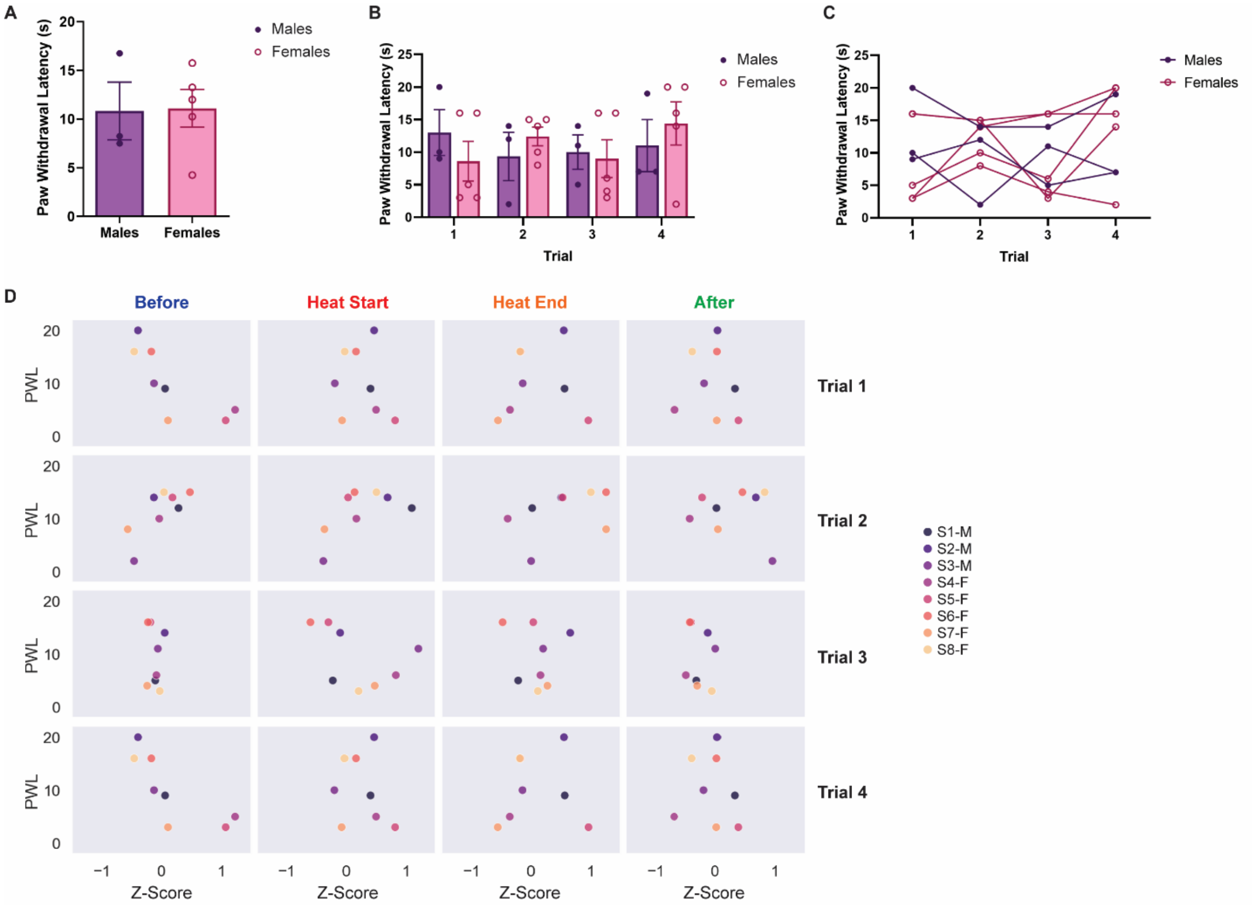

Supplemental Figure 1. Reflexive Behaviors of BNST^{CRF} Imaging Mice

(A) Thermal nociceptive sensitivity of male (purple; n = 3) and female (magenta; n = 5) CRF-Cre mice, as measured by the average paw withdrawal latency (PWL) following four trials of the Hargreaves test (unpaired t-test: $t(6) = 0.07921$, $p = 0.9394$).

(B) Progression of thermal nociceptive sensitivity by trial in male (purple) and female (magenta) subjects. Average PWL is indicated for each subject in trials 1-4 (Two-way mixed-model ANOVA with Sidak's post hoc: no Trial x Sex interaction [$F(3, 18) = 1.159$, $p = 0.3526$] or main effect of Trial [$F(2.498, 14.99) = 0.5867$, $p = 0.6041$] and Sex [$F(1, 6) = 0.0062$, $p = 0.9394$]).

(C) Trajectory of PWL across trials for individual male (purple) and female (magenta) subjects.

(D) Scatterplot of PWL and z-scores for individual subjects at each epoch and trial (S#-M = subject #, male; S#-F = subject #, female; see Figures 2A-2B for matching subject identities).

Data are shown as mean ±SEM. *$p < 0.05$; **$p < 0.01$; ***$p < 0.001$, ****$p < 0.0001$.

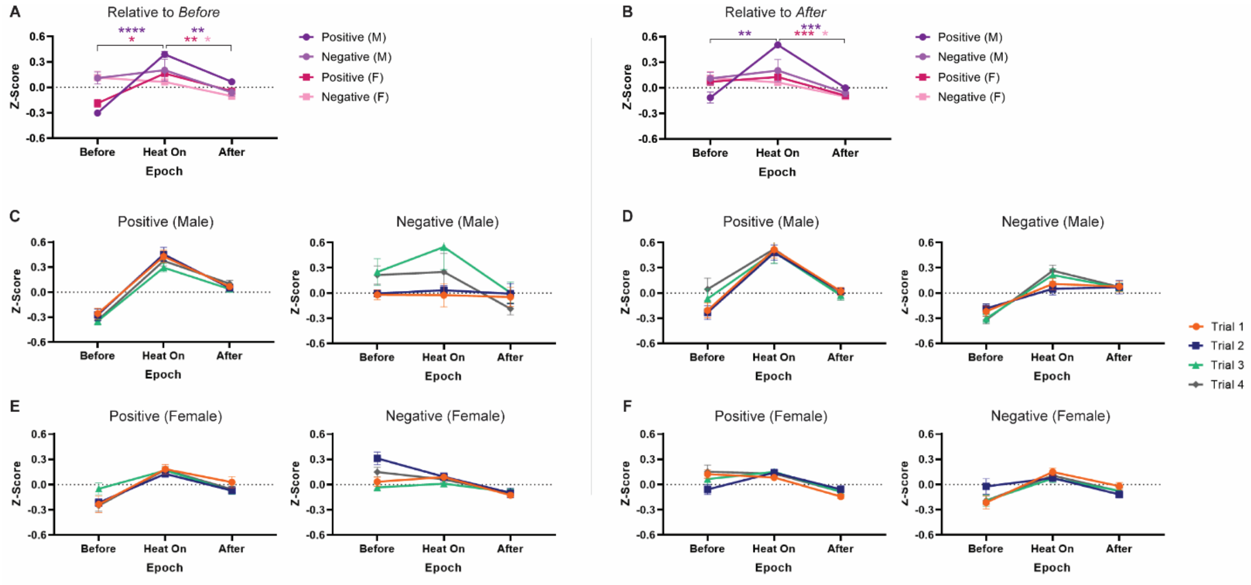

Supplemental Figure 2. BNST^{CRF} Activity by Response Type

(A-B) Average z-score of BNST^{CRF} neurons by heat response for male (purple) and female (magenta) subjects were determined relative to epochs surrounding the onset of **(A)** heat exposure [“Relative to *Before*”] and **(B)** paw withdrawal [“Relative to *After*”] across trials 1-4. Positive and negative responding cells are indicated by darker purple/magenta and lighter purple/magenta respectively (“Relative to *Before*”: Two-way repeated measures [RM] ANOVA with Tukey’s post hoc: Epoch x Responsivity interaction [F(6, 24) = 14.65, p < 0.0001], main effect of Epoch [F(1.816, 21.79) = 41.33, p < 0.0001], no main effect of Responsivity [F(3, 12) = 1.363, p = 0.3011]; “Relative to *After*”: Two-way RM ANOVA with Tukey’s post hoc: Epoch x Responsivity interaction [F(6, 24) = 9.417, p < 0.0001], main effect of Epoch [F(1.883, 22.60) = 34.55, p < 0.0001], no main effect of Responsivity [F(3, 12) = 1.567, p = 0.2486]).

(C-D) Comparison across trials of average BNST^{CRF} activity by response type (“Positive” vs. “Negative”) relative to epochs surrounding the onset of **(C)** heat exposure [“Relative to *Before*”] (“Relative to *Before*” / Positive: Two-way mixed-model ANOVA with Tukey’s post hoc: no Epoch x Trial interaction [F(6, 306) = 0.6838, p = 0.6628], main effect of Epoch [F(2, 111) = 71.69, p < 0.0001], no main effect of Trial [F(2.034, 207.4) = 2.415, p = 0.0909]; “Relative to *Before*” / Negative: Two-way mixed-model ANOVA with Tukey’s post hoc: no Epoch x Trial interaction [F(6, 72) = 1.167, p = 0.3338] or main effect of Epoch [F(2, 27) = 2.847, p = 0.0755] and Trial [F(2.166, 51.97) = 2.816, p = 0.0649]) and **(D)** paw withdrawal [“Relative to *After*”] (“Relative to *After*” / Positive: Two-way mixed-model ANOVA with Tukey’s post hoc: no Epoch x Trial interaction [F(6, 168) = 0.9217, p = 0.4808], main effect of Epoch [F(2, 90) = 23.83, p < 0.0001], no main effect of Trial [F(1.341, 75.12) = 0.04652, p = 0.8935]; “Relative to *After*” / Negative: Two-way mixed-model ANOVA with Tukey’s post hoc: Epoch x Trial interaction [F(6, 195) = 2.935, p = 0.0092] and main effect of Epoch [F(2, 96) = 26.51, p < 0.0001], no main effect of Trial [F(2.245, 145.9) = 0.1934, p = 0.8481]) in male subjects.

(E-F) Comparison across trials of average BNST^{CRF} activity by response type ("Positive" vs. "Negative") relative to epochs surrounding the onset of **(E)** heat exposure ["Relative to *Before*"] ("Relative to *Before*" / Positive: Two-way mixed-model ANOVA with Tukey's post hoc: Epoch x Trial interaction [F(6, 288) = 2,759, p = 0.0127], main effect of Epoch [F(2, 135) = 40.64, p < 0.0001], no main effect of Trial [F(2.522, 242.1) = 1.560, p = 0.2061; "Relative to *Before*" / Negative: Two-way mixed-model ANOVA with Tukey's post hoc: Epoch x Trial interaction [F(6, 228) = 3.226, p = 0.0046], main effect of Epoch [F(2, 141) = 8.364, p = 0.0004] and Trial [F(2.907, 220.9) = 4.907, p = 0.0028]) and **(F)** paw withdrawal ["Relative to *After*"] ("Relative to *After*" / Positive: Two-way mixed-model ANOVA with Tukey's post hoc: Epoch x Trial interaction [F(6, 297) = 2.775, p = 0.0122], main effect of Epoch [F(2, 123) = 10.94, p < 0.0001], no main effect of Trial [F(2.840, 281.1) = 1.210, p = 0.3058]; "Relative to *After*" / Negative: Two-way mixed-model ANOVA with Tukey's post hoc: no Epoch x Trial interaction [F(6, 258) = 1.953, p = 0.0729], main effect of Epoch [F(2, 114) = 14.36, p < 0.0001], no main effect of Trial [F(2.618, 225.1) = 0.7250, p = 0.5203]) in female subjects.

Data are shown as mean ±SEM. *p < 0.05; **p < 0.01; ***p < 0.001, ****p < 0.0001.

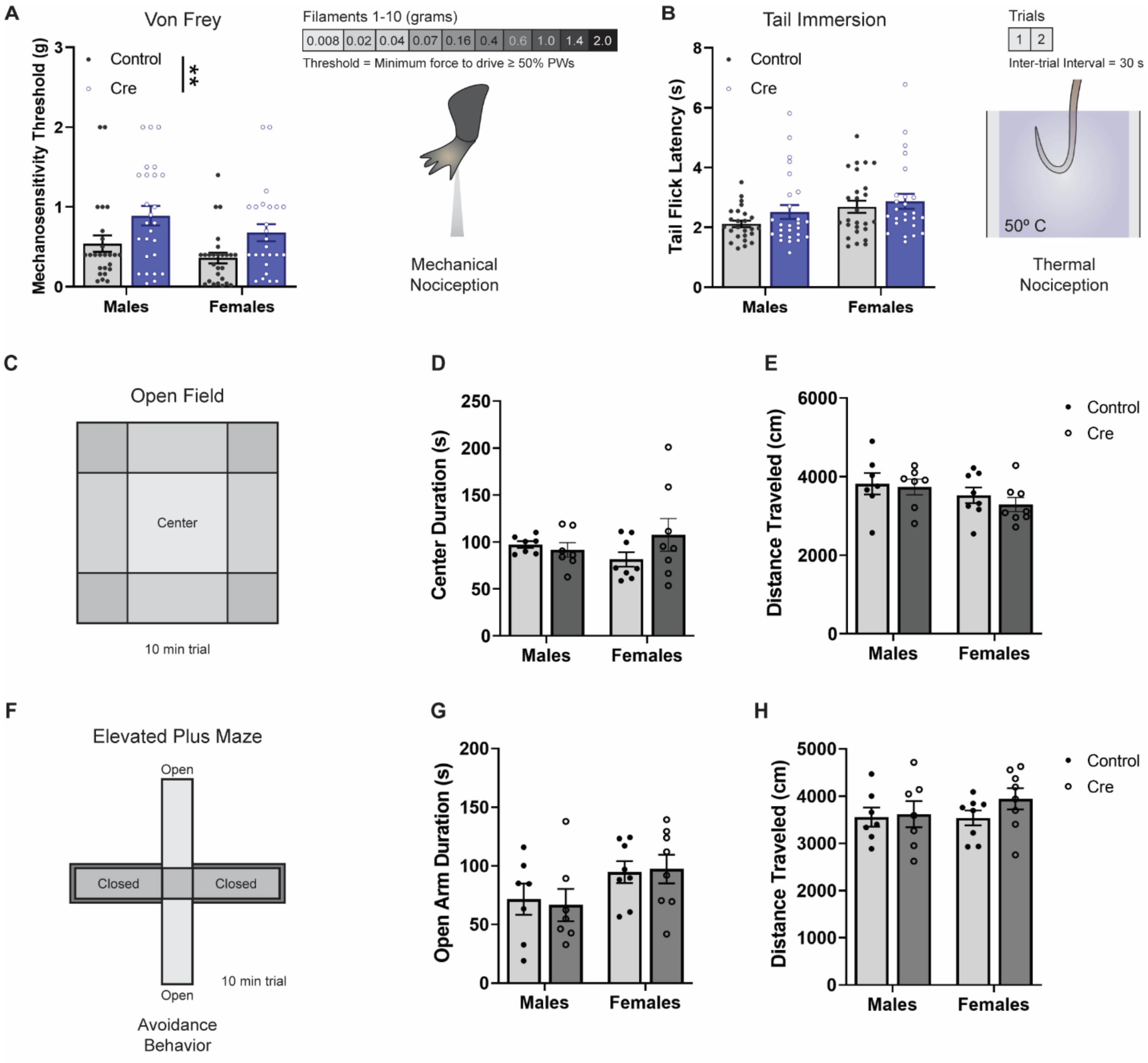

Supplemental Figure 3. CRF Deletion in BNST Affects Supraspinal Pain, Not Avoidance Behaviors

(A-B) Pain sensitivity of male (n = 25-26) and female (n = 25-26) Floxed-CRF mice with schematic of **(A)** Von Frey (Two-way ANOVA with Tukey's post hoc: no Sex x Virus interaction [F(1, 98) = 0.02228, p =0.8817] or main effect of Sex [F(1, 98) = 3.719, p = 0.0567]; main effect of Virus [F(1, 98) = 10.76, p = 0.0014]) and **(B)** tail immersion (Two-way ANOVA with Tukey's post hoc: no Sex x Virus interaction [F(1, 98) = 0.2772, p =0.5997] or main effect of Virus [F(1, 98) = 1.944, p = 0.1663]; main effect of Sex [F(1, 98) = 4.973, p = 0.0280]) tests.

(C) Schematic of open field test.

(D) Avoidance behaviors in male (n = 7) and female (n = 8) subjects, as measured by duration in the center (seconds) of the open field (Two-way ANOVA with Tukey's post hoc: no Sex x Virus interaction [F(1, 26) = 2.098, p = 0.1594], no main effect of Sex [F(1, 26) = 0.0001, p = 0.9911] and Virus [F(1, 26) = 0.8511, p = 0.3647]).

(E) Locomotor behaviors in male (n = 7) and female (n = 8) subjects, as measured by distance traveled (cm) in the open field (Two-way ANOVA with Tukey's post hoc: no Sex x Virus interaction [F(1, 26) = 0.1251, p = 0.7265], no main effect of Sex [F(1, 26) = 3.015, p = 0.0943] and Virus [F(1, 26) = 0.5665, p = 0.4584]).

(F) Schematic of elevated plus maze.

(G) Avoidance behaviors in male (n = 7) and female (n = 8) subjects, as measured by duration in the open arms (seconds) of the elevated plus maze (Two-way ANOVA with Tukey's post hoc: no Sex x Virus interaction [F(1, 26) = 0.0995, p = 0.7549], main effect of Sex [F(1, 26) = 4.908, p = 0.0357], no main effect of Virus [F(1, 26) = 0.0083, p = 0.9280]).

(H) Locomotor behaviors in male (n = 7) and female (n = 8) subjects, as measured by distance traveled (cm) in the elevated plus maze (Two-way ANOVA with Tukey's post hoc: no Sex x Virus interaction [F(1, 26) = 0.6221, p = 0.4374], no main effect of Sex [F(1, 26) = 0.4944, p = 0.4882] and Virus [F(1, 26) = 1.161, p = 0.2911]).

Data are shown as mean ±SEM. *p < 0.05; **p < 0.01; ***p < 0.001, ****p < 0.0001.

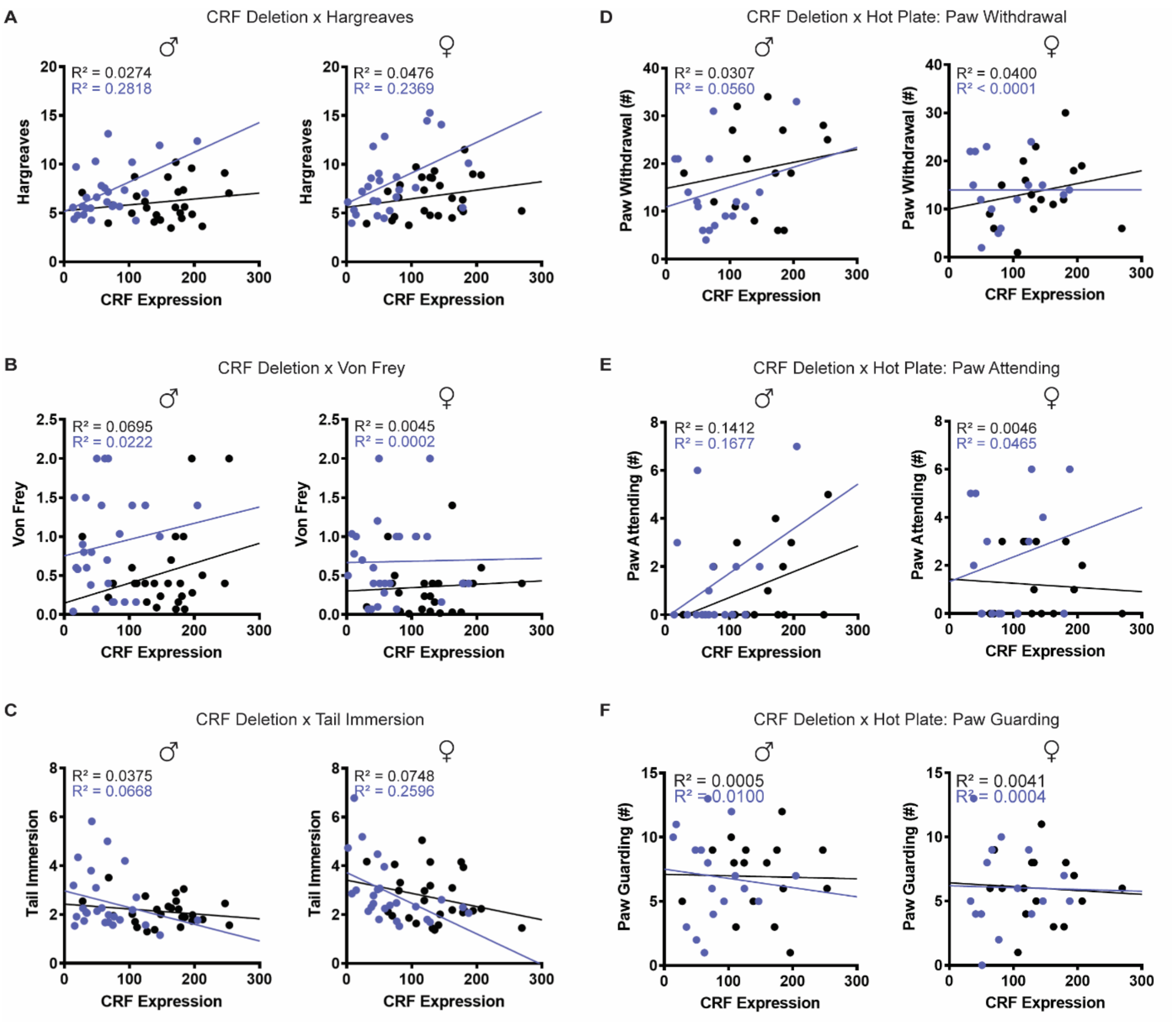

Supplemental Figure 4. Correlation Between CRF Expression in BNST and Pain-Related Behaviors

(A) Paw withdrawal latencies in response to thermal nociception as a function of CRF expression in the BNST of control- (black) or Cre- (blue) treated male (left; n = 25-26) and female (right; n = 25-26) Floxed-CRF mice.

(B) Paw withdrawal threshold in response to mechanism nociception as a function of CRF expression in the BNST of control- (black) or Cre- (blue) treated male (left; n = 25-26) and female (right; n = 25-26) subjects.

(C) Tail flick latency in response to thermal nociception as a function of CRF expression in the BNST of control- (black) or Cre- (blue) treated male (left; n = 25-26) and female (right; n = 25-26) subjects.

(D-F) Number of **(D)** paw withdrawal, **(E)** paw attending, and **(F)** paw guarding responses to thermal nociception in the prolonged hot plate as a function of CRF expression in the BNST of control- (black) or Cre- (blue) treated male (left; n = 16-17) and female (right; n = 15-17) subjects.

Data are shown using simple linear regression model. *p < 0.05; **p < 0.01; ***p < 0.001, ****p < 0.0001.

	Males		Females			
	CON	CRE	CON	CRE	p value	
	R^2	R^2	R^2	R^2	slopes	intercepts
Hargreaves	0.0274	0.2818	0.0476	0.2369	0.1031	0.0002
Von Frey	0.0695	0.0222	0.0045	0.0002	0.8014	0.0023
Tail Immersion	0.0375	0.0668	0.0748	0.2596	0.2914	0.1677
Hot Plate: Paw Withdrawal	0.0307	0.0560	0.0400	< 0.0001	0.9177	0.3756
Hot Plate: Paw Attending	0.1412	0.1677	0.0046	0.0465	0.5101	0.1412
Hot Plate: Paw Guarding	0.0005	0.0100	0.0041	0.0004	0.9928	0.7165

Table 1. Correlation Values of CRF Expression x Pain-Related Behaviors

Goodness-of-fit relationship between CRF expression in the BNST and pain-related behaviors, as determined by the coefficient of determination (R^2) in a simple linear regression model. The slopes and intercepts were compared by virus and sex: M-CON, M-CRE, F-CON, F-CRE (Two-way ANCOVA; Hargreaves: Slopes [F(3, 94) = 2.119, p = 0.1031], Intercept [F(3, 97) = 7.129, p = 0.0002]; Von Frey: Slopes [F(3, 94) = 0.3331, p = 0.8014], Intercept [F(3, 97) = 5.183, p = 0.0023]; Tail Immersion: Slopes [F(3, 94) = 1.264, p = 0.2914], Intercept [F(3, 97) = 1.721, p = 0.1677]; Hot Plate: Paw Withdrawal: Slopes [F(3, 56) = 0.1678, p = 0.9177], Intercept [F(3, 59) = 1.054, p = 0.3756]; Hot Plate: Paw Attending: Slopes [F(3, 56) = 0.7800, p = 0.5101], Intercept [F(3, 59) = 1.890, p = 0.1412]; Hot Plate: Paw Guarding: Slopes [F(3, 56) = 0.0302, p = 0.9928], Intercept [F(3, 59) = 0.4525, p = 0.7165]).

Data are shown as R^2 and p values. *p < 0.05; **p < 0.01; ***p < 0.001, ****p < 0.0001.
